# Temporal associations between incident physical health problems/sensory impairments and challenging behaviours in people with intellectual disabilities: a population-based longitudinal cohort study of primary care in England

**DOI:** 10.1136/bmjopen-2025-111117

**Published:** 2026-07-03

**Authors:** Elliot Millington, Ewelina Rydzewska, Andrew Jahoda, Richard P Hastings, Anne MacDonald, Michael Fleming, Caroline Richards, Amanda Gillooly, Elita Smiley, Umesh Chauhan, Christine Pacatti, Craig Melville

**Affiliations:** 1 School of Health and Wellbeing, University of Glasgow, Glasgow, UK; 2 School of Health in Social Science, The University of Edinburgh, Edinburgh, UK; 3 Intellectual Disabilities Research Institute (IDRIS), University of Birmingham, Birmingham, UK; 4 School of Psychology, University of Birmingham, Birmingham, UK; 5 NHS Greater Glasgow and Clyde, Glasgow, UK; 6 School of Health, University of Lancashire, Preston, UK

**Keywords:** Primary Health Care, Longitudinal studies, Multimorbidity, Electronic Health Records, Developmental neurology & neurodisability, Suicide & self-harm

## Abstract

**Abstract:**

**Objectives:**

To determine whether the onset of physical health problems/sensory impairments is associated with incident challenging behaviours.

**Design:**

A retrospective, population-based cohort study using longitudinal data from primary care records. HRs were estimated using Cox proportional hazards models accounting for recurrent events and time-varying exposures.

**Setting:**

UK primary care data sourced from the Clinical Practice Research Datalink (CPRD) Aurum and Gold databases, covering over 850 000 person-years between 2009 and 2019.

**Participants:**

166 989 individuals with recorded intellectual disabilities were included in the cohort.

**Primary outcome measures:**

Incident identification of challenging behaviours before or after a recorded incident of physical health problems/sensory impairment. Physical health problems/sensory impairments assessed included constipation, epilepsy, pain, visual impairment, hearing impairment, bowel incontinence, urinary incontinence and sleep problems.

**Results:**

21.21% (n=35 415) of the cohort had challenging behaviour recorded at least once in primary care records over the 11-year study period, equating to an incidence rate of 0.10 per person-year. 40.9% of episodes of challenging behaviour were associated with an incident physical health problem/sensory impairment. All eight physical health problems/sensory impairments were significantly associated with higher HRs for challenging behaviours after full adjustment for demographic and mental health covariates. These associations held across multiple sensitivity analyses. The strongest associations were found for bowel incontinence (HR=2.24; 95% CI 2.01 to 2.50), urinary incontinence (HR=1.93; 95% CI 1.77 to 2.11), constipation (HR=1.89; 95% CI 1.74 to 2.05) and sleep problems (HR=1.74; 95% CI 1.58 to 1.90).

**Conclusions:**

This is the first longitudinal study to establish a temporal association between the onset of physical health problems/sensory impairments and challenging behaviours in people with intellectual disabilities. These findings highlight the need for proactive identification and management of physical health problems/sensory impairments as part of assessment processes to prevent or reduce the impact of challenging behaviours.

**Strengths and limitations of this study**

- This study is the largest longitudinal study to date examining challenging behaviours in adults with intellectual disabilities and the only study looking at temporal associations with physical health problems/sensory impairments.
- The data were collected in primary care, maximising the potential relevance to healthcare services.
- Primary care coding likely under-identifies challenging behaviours as only the most severe cases are seen in clinical settings.
- Over 70% of patients had missing data on intellectual disability severity.

## Introduction

 Population-based studies suggest that around 20%–30% of individuals with intellectual disabilities experience challenging behaviours.^1^,^3^ Challenging behaviours include aggression, property destruction and self-injurious behaviour.^4^ Many people with intellectual disabilities and challenging behaviours are prescribed psychotropic medication.^5^ Challenging behaviours can have a negative impact on the education,^6^ quality of life^7^ and social inclusion^8^ of people with intellectual disabilities.

**Figure 1:**
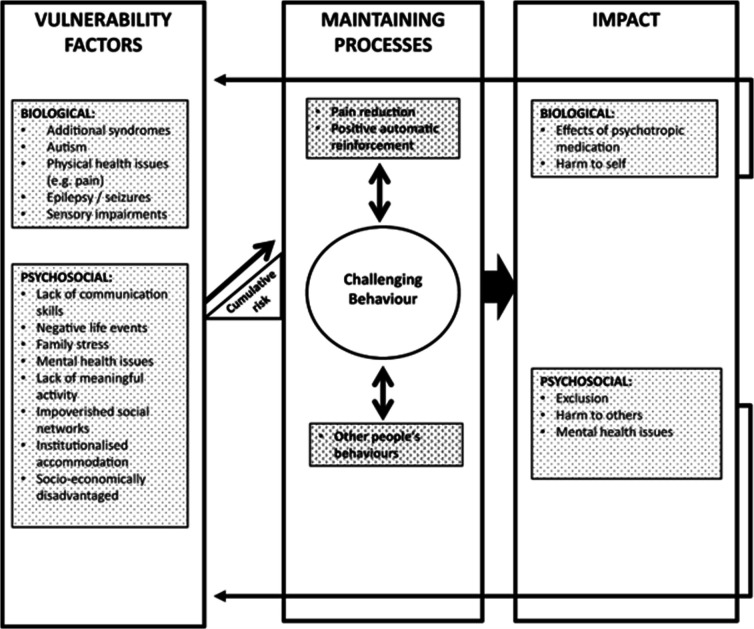
A framework for challenging behaviours (Bowring *et al*^12^—reproduced in accordance with Creative Commons Attribution 4.0 International License).

Longitudinal studies have reported that challenging behaviours typically persist over time.^9 10^ However, for some people, challenging behaviours remit or fluctuate over time^9 11^ and can present for the first time at any age, including adulthood.^9 11^ Potential correlates and risk factors associated with the onset or persistence of challenging behaviours have been explored in research. Bowring *et al*^12^ have synthesised this evidence on correlates and risk factors to propose a framework model for challenging behaviours. This framework for understanding challenging behaviours is reproduced in figure 1.

Of greatest relevance to exploring the fluctuating course and onset of challenging behaviours over time is the biopsychosocial model for vulnerability factors that are proposed to cumulatively increase the risk of challenging behaviours.^12^ There has already been considerable research exploring the relationships between mental health issues and challenging behaviours,^13^ shown in the framework. To examine a separate component of the framework, the study reported here focuses on the proposal that physical health problems/sensory impairments can act as biological vulnerability factors for challenging behaviours. Since physical health problems/sensory impairments can be reliably diagnosed and effectively treated, they may be of relevance to clinical services supporting people with intellectual disabilities and challenging behaviours, and health improvement initiatives more broadly.

A systematic review of studies investigating the association of physical health problems, sensory and other impairments with challenging behaviours in populations with intellectual disabilities identified 45 cross-sectional studies.^14^ These studies looked at general medical conditions, motor impairment, epilepsy, sensory impairment, gastrointestinal (GI) disease, sleep disorders, dementia and other conditions. Among the identified studies, there were only four high-quality and seven well-conducted observational studies. The authors concluded from the narrative synthesis that there was evidence to support an association between challenging behaviours and urinary incontinence, pain related to cerebral palsy and chronic sleep problems.

There have also been several evidence syntheses exploring the association of individual physical health problems with challenging behaviours. A systematic review focused on sleep problems reported that six out of a total of seven included cross-sectional studies reported a significant association between sleep and challenging behaviours.^15^ Blickwedel *et al*^16^ concluded from a systematic review of five cross-sectional studies that there was minimal evidence supporting an association between epilepsy and challenging behaviours. However, a more recent systematic review and meta-analysis looking at the evidence for an association between epilepsy and challenging behaviours reported a very small increase in challenging behaviours in individuals diagnosed with epilepsy compared with individuals without epilepsy in a meta-analysis of a sub-set of 10 cross-sectional studies.^17^

While the results from these four evidence synthesis studies are of interest, the internal validity of the findings are threatened by the cross-sectional design of every (100%) study included in the meta-analyses. The studies included in the four syntheses^14^,^17^ examined whether the participants have experienced both the physical health problems/sensory impairments and challenging behaviours. However, they were not able to examine whether there was a temporal association between the onset of physical health problems/sensory impairments and challenging behaviours. For example, the onset of epilepsy could have been 10 years before the onset of challenging behaviours. To overcome this limitation, in the present study we examined the relationship in time between physical health problems/sensory impairments and challenging behaviours in a longitudinal, population-based cohort of people with intellectual disabilities in primary care. If this study finds that physical health problems/sensory impairments are commonly experienced biological vulnerability factors for incident challenging behaviours, this will impact clinical practice by creating a greater focus on physical health and sensory impairments. To explore this further, we examined two research questions:

1. What is the incidence of reported challenging behaviours in people with intellectual disabilities?
2. Is there an increased incidence of challenging behaviours in relation to the onset of physical health problems/sensory impairments in people with intellectual disabilities?

## Methods

### Patient and public involvement

The Scottish Learning Disabilities Observatory at the University of Glasgow initiated this study following concerns from people with intellectual disabilities, their families and carers. The Observatory operates with a steering group that includes both people with intellectual disabilities and third-sector organisation partners. Study results will be disseminated in accessible formats to people with intellectual disabilities and their families through the Observatory website, social media channels and newsletters.

### Data source

This study used primary care data from the Aurum and Gold databases maintained by the Clinical Practice Research Datalink (CPRD).^18^ In the UK, almost all residents are registered with a general practitioner (GP) practice, which provides free healthcare on behalf of the National Health Service (NHS). CPRD contains data from participating GP practices in the UK linked to GP practice-level Index of Multiple Deprivation. CPRD Aurum contains data from practices which use *EMIS Web* software for their data management, and CPRD Gold contains data from practices which use the *Vision* software for the same purpose. 15.29% of UK practices participate in the CPRD Aurum, which served 19.75% of the UK population as current patients at the time of extraction. CPRD Gold contained 4.40% of practices and 4.52% of the UK population. Prior studies have concluded that records held by CPRD are broadly representative of the UK population, with a slight overrepresentation of ethnic minorities.^19 20^

### Study design

This paper describes a retrospective cohort study of people with intellectual disabilities, using data between 1 January 2009 and 31 December 2019. Complete records were requested for any person with a record of intellectual disabilities in the primary care data, as determined using read codes (see codes used in online supplemental file 1). The definition of intellectual disabilities was based on codes used by the NHS Primary Care Quality and Outcomes Framework for the development of intellectual disabilities registers, following the precedent of previous studies using CPRD data.^5^

The cohort entry date for each participant was defined as the latest of the following: the first record of intellectual disabilities, the date of registration with a participating GP practice or 1 January 2009.

### Exposures

The exposures in this study were physical health problems/sensory impairments that are commonly identified as vulnerability factors relevant to the onset of incident challenging behaviours. Physical health problems/sensory impairments were included if they have been reported in previous evidence syntheses to be associated with an increased risk of challenging behaviours by people with intellectual disabilities^14^,^17^ or observed as associated with the onset of challenging behaviours in clinical practice. Based on the consensus of the research team about the existing evidence base and the consensus of the clinicians involved in the study, the physical health problems/sensory impairments included in the analyses were:

- constipation
- epilepsy
- pain
- visual impairment
- hearing impairment
- bowel incontinence
- urinary incontinence
- sleep problems.

In keeping with previous studies,^16 17^ and to improve the validity of the epilepsy construct used in this study, we focused on epilepsy diagnoses, rather than febrile and non-febrile seizures. This focus on epilepsy diagnoses has the effect of increasing the homogeneity of the epilepsy caseness by requiring a clinical diagnosis, and removing non-epilepsy aetiologies.

There is evidence that gastro-oesophageal reflux disorder (GORD) is more commonly experienced by people with intellectual disabilities.^21^ Individuals with intellectual disabilities and GORD seldom present with upper GI symptoms of GORD.^22^ Instead, the presentation of challenging behaviours in people with intellectual disabilities is often identified as possible GORD and treated empirically with medication. People with intellectual disabilities are also very unlikely to undergo confirmatory diagnosis of GORD via upper GI endoscopy due to the invasive nature of the procedure. Therefore, we did not include GORD as one of the physical health problems/sensory impairments because in clinical practice there is not sufficient discriminatory validity between GORD and challenging behaviours to reliably explore potential links between the onset of GORD and challenging behaviours. However, some episodes of incident GORD may be included in the pain diagnoses.

The first clinical record of individual physical health problems/sensory impairments was considered an incident episode. Since the included physical health problems/sensory impairments can have a recurring clinical pattern, further incident episodes of each condition were also included in the analysis. These were defined as where a code was not linked to an ongoing episode of care, and the same code had not been recorded in the previous 12 months.

### Study outcomes

The primary outcome considered by this study was the reporting of challenging behaviours, recorded in primary care records. The list of codes for challenging behaviours considered for this study was adapted from Sheehan *et al*^5^ and is listed in online supplemental file 1.

### Covariates

We included sex, age, ethnicity, GP practice area level socioeconomic deprivation, autism spectrum diagnosis and mental health conditions as potential covariates.

For descriptive analyses, patients were categorised by age at study entry into 8 groups (<18, 18–29, 30–39, 40–49, 50–59, 60–69, 70–79 and 80+ years). For inferential analyses, age was treated as a continuous variable.

We categorised individuals into five ethnic groups: Asian, Black, mixed, other, White or unknown, based on recorded values in their CPRD records.

Area-level socioeconomic deprivation measured at the GP practice level was coded using quintiles of the Index of Multiple Deprivation (IMD): quintile 1 (low deprivation) to quintile 5 (high deprivation). Socioeconomic deprivation was assessed using the 2019 English IMD, which considers characteristics of the small geographic area, such as income, employment and education levels, to create a composite score.

Autism spectrum diagnosis was included as a potential covariate because of the evidence showing that individuals with intellectual disabilities and a coexisting autism spectrum condition are at increased risk of challenging behaviours.^23^ Severity of intellectual disabilities has also been shown to have a significant association with challenging behaviours.^3^ However, data on the severity level of intellectual disabilities were only available for less than 30% of the cohort, so severity of intellectual disabilities was excluded from the primary analysis. Data on severity of intellectual disabilities were systematically more likely to be missing from records for participants with mild intellectual disabilities.

A consensus decision was made to include anxiety disorders, bipolar disorder, major depressive disorder and schizophrenia as potential covariates. These four mental health conditions are commonly experienced by individuals with intellectual disabilities and have been shown in population-based epidemiological studies to be associated with challenging behaviours.^24^ The onset of dementia can also be associated with challenging behaviours. We did not include dementia as a potential covariate because challenging behaviours in dementia are typically conceptualised as a distinct syndrome, called the behavioural and psychological symptoms of dementia.^25^

All read codes used for the covariates are included in online supplemental file 3.

### Data analyses

Analyses were conducted using R V.4.4.2^26^ and the *Survival* package.^27 28^

The number of recorded incident challenging behaviours was counted for the entire sample and then stratified by age group, sex, socioeconomic deprivation and presence of an autism spectrum condition. These values were then used along with the amount of time cohort members spent in the study to calculate the incidence rate of challenging behaviours per person-year.

To assess the risk of developing challenging behaviours associated with incident physical health conditions, HRs were estimated using the Andersen-Gill extension of the Cox proportional hazards model with separate models fitted for each individual physical health condition. All models accounted for clustering at the practice level and repeated measures within the individual using robust variance estimation.

The challenges surrounding the recognition of physical health problems/sensory impairments experienced by individuals with intellectual disabilities have been described in detail previously.^29^ These difficulties recognising physical health problems/sensory impairments often result in prolonged periods during which such conditions remain unrecognised and unmanaged. During this time, challenging behaviours may emerge as the initial presenting issue before the underlying health problem is identified. To account for this temporal uncertainty, each incident physical health problem created an exposure period extending 12 months before and after the recorded clinical event, bounded by study entry and exit dates. This 12-month window was selected based on the consensus clinical judgement of the senior clinicians, from multidisciplinary professional backgrounds, in the research team regarding typical diagnostic delays and confirmed through model diagnostics assessing proportional hazards assumptions. Sensitivity analyses using alternative windows of 1, 3 and 6 months are reported in online supplemental file 4.

When individuals experienced multiple physical health conditions with overlapping exposure windows, a series of mutually exclusive time intervals were created for the analysis. Each interval was characterised by the combination of conditions present during that period. For example, an interval might be classified as having sleep problems only, constipation exposure only, both exposures simultaneously or neither. Individuals contributed person-time from study entry to exit, with their exposure status updating at each clinical event date. After experiencing a challenging behaviour event, individuals remained at risk for subsequent events. The models were adjusted for demographic variables (age, sex, ethnicity, deprivation and the presence of an autism spectrum condition) and then a final model was adjusted for demographic variables and incident mental health diagnoses treated as time-varying covariates using the same methodology as physical health conditions, with each mental health condition modelled separately with exposure periods extending 12 months before and after each event date.

Violations of the assumption of proportional hazards were assessed using a combination of formal tests of Schoenfeld residuals and graphical methods. In response to these violations, models which accounted for demographic variables were stratified by sex, ethnicity, deprivation and the presence of an autism spectrum condition. Patterns of missingness were assessed by comparing the demographic and clinical characteristics between those individuals with and without complete data, the results of which can be found in online supplemental file 5. These analyses indicated that missingness was associated with several observed covariates, consistent with the data being Missing At Random. As such, each analysis presented here was conducted using a complete case approach that only includes participants with no missing data. As a sensitivity analysis, modelling was completed that included only participants with available data on severity of intellectual disabilities (online supplemental file 6). As another sensitivity analysis, an alternative dataset was created using multiple imputation with chained equations for those with missing data for their ethnicity, socioeconomic deprivation and severity of intellectual disabilities (online supplemental file 7). The participants’ GP practice, sex, ethnicity, socioeconomic deprivation of their local area, age at study entry and presence of the diagnostic outcomes in this study were each used to inform the imputations.

## Results

### Cohort members

This study included records from 166 989 people identified as having intellectual disabilities who collectively contributed 854 133 person-years or a mean of 5.8 years per person. The mean age of the cohort at entry to the study was 33.1 (SD 19.4). Further demographic characteristics of this cohort can be found in table 1. Less than five people with indeterminate sex were present in the data but were excluded from the analysis to reduce disclosure risks.

**Table 1:** Demographic characteristics of cohort

Demographics	n	Percentage of sample
Sex		
Female	69 297	41.50
Male	97 692	58.50
Ethnicity		
White	68 984	41.31
Asian	9739	5.83
Black	7591	4.55
Mixed	3038	1.82
Other	1640	0.98
Unknown	75 997	45.51
GP practice-level IMD		
1	21 405	12.82
2	25 947	15.54
3	31 744	19.01
4	39 842	23.86
5	47 897	28.68
Unknown	154	0.09
Physical health problems/sensory impairments		
Constipation	20 989	12.57
Epilepsy	26 519	15.88
Pain	66 855	40.04
Visual impairment	12 869	7.71
Hearing impairment	6829	4.09
Bowel incontinence	4067	2.44
Urinary incontinence	11 180	6.70
Sleep problems	34 240	20.50
Mental health conditions		
Anxiety	14 534	8.70
Bipolar disorder	1619	0.97
Major depression	13 027	7.80
Schizophrenia	4857	2.91
ID severity level		
Mild	18 263	10.94
Moderate	17 593	10.54
Severe	12 329	7.38
Profound	1162	0.70
Unknown	117 642	70.45
Autism spectrum condition	30 362	18.18

GP, general practitioner; ID, intellectual disability; IMD, Index of Multiple Deprivation.

### Incidence of challenging behaviours

A total of 87 938 incident records of challenging behaviours were listed in GP records, leading to a reported incidence rate of 0.10 per person-year. 35 415 participants or 21.21% of the total sample had one or more recorded challenging behaviours over the study period. The number of separate incident challenging behaviours and their incidences by demographic subgroups can be found in table 2.

**Table 2:** Incidence of challenging behaviours stratified by subgroups

	Challenging behaviour events	Person-years	Rate per person-year
Sex			
Female	37 077	277 109	0.13
Male	50 861	382 808	0.13
Age group			
<18	13 551	103 542	0.13
18–29	21 367	151 404	0.14
30–39	13 363	106 767	0.12
40–49	17 089	138 978	0.12
50–59	12 157	90 481	0.13
60–69	6956	49 687	0.14
70–79	2821	16 135	0.17
80+	634	2923	0.22
Ethnicity			
White	43 570	334 136	0.13
Asian	5262	41 919	0.13
Black	3517	25 097	0.14
Multiple	1551	11 213	0.14
Other	615	4365	0.14
IMD			
1	10 101	76 136	0.13
2	13 942	105 923	0.13
3	18 232	138 990	0.13
4	20 359	151 378	0.13
5	25 248	187 151	0.14
ID severity level			
Mild	12 594	92 751	0.14
Moderate	15 250	121 537	0.12
Severe	13 894	112 960	0.12
Profound	1325	11 197	0.12
Autism			
No	66 019	496 960	0.13
Yes	21 919	162 957	0.14

ID, intellectual disability; IMD, Index of Multiple Deprivation.

The percentage of the total sample experiencing incident challenging behaviours is similar to the findings on the only other population-based study that examined incidence over a 2-year period.^11^

### Physical health problems/sensory impairments and challenging behaviours

A total of 106 617 or 63.85% of the people with intellectual disabilities in this study had a record of at least one of the incident physical health problems/sensory impairments over the study period. The number of incident physical health problems/sensory impairments, the incidence per person-year, the number of unique individuals experiencing these events, and the proportion of the sample experiencing the physical health problems/sensory impairments—with the cohort categorised by the presence of a record of challenging behaviour—are presented in table 3.

**Table 3:** Incidence of clinical events over study period by record of challenging behaviours in primary care records

	Challenging behaviour present			No challenging behaviour		
	Events	Incidence	n (%)	Events	Incidence	n (%)
Constipation	24 682	0.12	8057 (26.6)	31 442	0.07	12 786 (16.8)
Epilepsy	85 783	0.40	8835 (29.1)	126 859	0.29	17 258 (22.6)
Pain	89 126	0.42	18 973 (62.5)	182 850	0.42	47 568 (62.4)
Visual impairment	10 819	0.05	5427 (17.9)	12 689	0.03	7076 (9.3)
Hearing impairment	5952	0.03	2648 (8.7)	7215	0.02	3978 (5.2)
Bowel incontinence	4054	0.02	2085 (6.9)	3131	0.01	1900 (2.5)
Urinary incontinence	11 737	0.06	5035 (16.6)	12 017	0.03	5970 (7.8)
Sleep problems	42 662	0.20	13 080 (43.1)	58 950	0.14	20 683 (27.1)

Note. Incidence is presented as events per person-year.

The results in table 3 show that constipation, epilepsy, visual impairment, hearing impairment, bowel incontinence, urinary incontinence and sleep problems occur more frequently in the subgroup categorised as having experienced one or more incident episodes of challenging behaviour. Pain had a much higher prevalence than the other physical health problems/sensory impairments and was similar in both subgroups, being recorded in over 60% of the cohort with and without challenging behaviours.

40.9% of episodes of challenging behaviours were associated in time with an incident physical health problem/sensory impairment. The results from the Cox proportional hazard models to examine temporal associations between incident physical health problems and challenging behaviours, are reported in table 4.

**Table 4:** Cox proportional hazards regression models

Predictor	Unadjusted		Demographically adjusted		Fully adjusted	
	HR	P value	HR	P value	HR	P value
Constipation	2.273 (2.103 to 2.457)	<0.001***	2.080 (1.899 to 2.278)	<0.001***	1.890 (1.742 to 2.052)	<0.001***
Epilepsy	1.691 (1.555 to 1.840)	<0.001***	1.619 (1.468 to 1.786)	<0.001***	1.583 (1.452 to 1.727)	<0.001***
Pain	1.596 (1.498 to 1.702)	<0.001***	1.458 (1.343 to 1.583)	<0.001***	1.321 (1.230 to 1.420)	<0.001***
Visual impairment	1.856 (1.705 to 2.021)	<0.001***	1.721 (1.561 to 1.898)	<0.001***	1.651 (1.511 to 1.803)	<0.001***
Hearing impairment	1.964 (1.744 to 2.213)	<0.001***	1.783 (1.603 to 1.982)	<0.001***	1.751 (1.586 to 1.932)	<0.001***
Bowel incontinence	2.543 (2.292 to 2.822)	<0.001***	2.323 (2.063 to 2.616)	<0.001***	2.241 (2.007 to 2.504)	<0.001***
Urinary incontinence	2.310 (2.112 to 2.527)	<0.001***	2.098 (1.899 to 2.319)	<0.001***	1.930 (1.768 to 2.106)	<0.001***
Sleep problems	1.992 (1.835 to 2.161)	<0.001***	1.903 (1.713 to 2.113)	<0.001***	1.735 (1.582 to 1.902)	<0.001***

Demographically adjusted models included age as a predictor and were stratified by sex, ethnicity and IMD. Fully adjusted models included the demographic adjustments, as well as diagnoses of anxiety, bipolar disorder, major depression and schizophrenia as predictors, and were additionally stratified by diagnoses of autism.

* p<0.05, **p<0.01, ***p<0.001.

IMD, Index of Multiple Deprivation.

The unadjusted model in table 4 shows that there was a significantly higher risk of challenging behaviours in the 12 months before and after the onset of all eight physical health problems/sensory impairments. Adjusting for the demographic variables (age, sex, ethnicity, deprivation and the presence of an autism spectrum condition) had a minimal impact on the HRs. As expected, in the fully adjusted model controlling for mental health conditions that were listed in the dataset at the time of the incident health problem reduced the HRs for all physical health problems/sensory impairments.

### Sensitivity analyses

The sensitivity analyses, in which we analysed only participants with available data on severity of intellectual disabilities (online supplemental file 6) and used multiple imputation for missing data on ethnicity, socioeconomic deprivation and severity of intellectual disabilities (online supplemental file 7) showed a similar pattern of results to those of the primary analysis. However, compared with the HRs in the primary analysis (table 4), the size of the HRs decreased when we analysed the reduced sample of participants with data on severity of intellectual disabilities (online supplemental table S10). This effect may be partially attributable to the reduced sample size and the introduction of a selection bias when restricting the cohort to participants with available data on severity of intellectual disabilities.

The significantly increased risk of challenging behaviours persisted in the sensitivity analyses that reduced the temporal window to 1, 3 and 6 months before and after the physical health problems/sensory impairments (online supplemental file 4).

## Discussion

This large, population-based cohort study investigated the temporal relationship between incident physical health problems/sensory impairments and the onset of reported challenging behaviours among people with intellectual disabilities using primary care data from CPRD Aurum and Gold databases. We found that over one-fifth of individuals (21.21%) had at least one recorded episode of challenging behaviour during the 11-year study period, with an overall incidence rate of 0.10 per person-year. Over 40% of recorded episodes of challenging behaviours were associated in time with an incident physical health problem/sensory impairment and all eight physical health problems/sensory impairments examined were significantly associated with an increased risk of challenging behaviours in the 12 months surrounding their first clinical event, even after adjusting for key demographic and mental health confounders. Sensitivity analyses found that temporal relationship between physical health problems/sensory impairments and challenging behaviours was consistent when the temporal window was reduced to 1, 3 and 6 months.

### Comparison with previous studies

There has only been one previous, population-based study reporting the incidence of challenging behaviours experienced by adults with intellectual disabilities. A Scottish study followed up 651 adults with intellectual disabilities over 2 years and reported that 4.6% of participants experienced an incident episode of challenging behaviours.^11^ However, the number of incident episodes of challenging behaviours in the 2-year period was too small to explore associations with physical health problems or sensory impairments. Comparison with this study is difficult because the authors did not report incidence rate per person-year. Both studies illustrate that challenging behaviours fluctuate over time, potentially impacting on the well-being and quality of life of adults with intellectual disabilities.^7^

Our findings provide the first evidence from a longitudinal study design to support predictions from a biopsychosocial model perspective that proposes that physical health problems/sensory impairments can act as biological vulnerability factors for the development of challenging behaviours (eg, Bowring *et al*^12^). As described in previously published meta-analyses,^14^,^17^ the evidence supporting a link between physical health problems/sensory impairments came from cross-sectional studies, which were unable to examine a temporal relationship between physical health problems/sensory impairments and challenging behaviours. The results provided here emphasise the need for a robust, routine assessment of physical health problems/sensory impairments as part of the assessment of any person presenting with a change in challenging behaviours.

New episodes of all eight of the physical health problems/sensory impairments investigated in this study were associated with the onset of challenging behaviours. The generality of the association across physical health problems/sensory impairments suggests that the challenging behaviours may in part communicate or be a direct response to discomfort, distress or pain that individuals are experiencing due to unrecognised physical health problems/sensory impairments.^30^ The finding that the higher risk of challenging behaviours was unchanged for 1, 3, 6 and 12-month time windows before and after the recorded diagnoses of provides further evidence that physical health problems/sensory impairments are commonly unrecognised for long periods and often present alongside challenging behaviours. This is a crucial issue to consider because the delayed diagnosis and management of significant physical health problems/sensory impairments has been linked to the premature death of people with intellectual disabilities.^29^

The difficulties recognising physical health problems/sensory impairments in adults with intellectual disabilities have led some countries to introduce annual health checks for adults with intellectual disabilities. There is some evidence in particular that annual health checks lead to increased recognition of constipation, sensory impairments, and pain.^31 32^ National clinical guidelines^33^ and standards^34^ in England and Wales recommend annual health checks as a preventative measure to address challenging behaviour. Adults with intellectual disabilities found on risk indices to have an increased risk for challenging behaviour (eg, Bowring *et al*^3^) might be a particular priority for more regular health screening as a preventative measure. As an adjunct to annual health checks, there is a need to train carers and support staff working with people with intellectual disabilities to recognise signs of undiagnosed physical health problems/sensory impairments,^35^ and to train mainstream healthcare professionals in the assessment and management of physical health problems/sensory impairments experienced by adults with intellectual disabilities.^36^

Challenging behaviours may represent communication and/or response to discomfort, distress or pain as a general mechanism. However, there may be other potential mechanisms linking specific physical health problems with the onset of challenging behaviours. The links between sleep problems and daytime challenging behaviours experienced by children^37^ and adults^38^ with intellectual disabilities were described in studies several decades ago. There have been relatively few studies exploring the mechanisms underlying the relationship between sleep problems and challenging behaviours in individuals with intellectual disabilities. One review included seven case studies, six of which had one participant and one study that included two participants.^39^ The author concludes that sleep deprivation leads to challenging behaviours through negative reinforcement operant mechanisms. Significantly more research has explored the mechanisms underlying the complex relationship between sleep problems and aggressive behaviours experienced by individuals who do not have intellectual disabilities.^40^ For example, Krizan and Herlache^41^ proposed separate affective (eg, increased pain sensitivity), cognitive (eg, paranoid interpersonal attributions) and impulse control (eg, reduced self-control) pathways to explain the link between sleep problems and aggression. More recently, researchers have focused on emotional dysregulation^42^ being a potential pathway between sleep problems and aggression. This broader evidence base can be used to inform research into potential mechanisms underlying the relationships between sleep problems and aggression, or other challenging behaviours, experienced by people with intellectual disabilities.

### Strengths and limitations

Longitudinal studies involving people with intellectual disabilities are rarely published. This is the largest longitudinal analysis to date examining the incidence of challenging behaviours in adults with intellectual disabilities and the only study examining temporal associations between incident physical health problems/sensory impairments and challenging behaviours in individuals with intellectual disabilities. The use of routinely collected primary care data embeds this study in real-world clinical services, maximising the potential relevance of the findings to healthcare services.

Demonstrating that the higher risk of challenging behaviours persists when 1, 3, 6 and 12-month time windows were used supports the validity of the findings and provides important evidence that challenging behaviours are often the first presentation of physical health problems/sensory impairments.

Despite these strengths, several limitations must be acknowledged. First, coding in primary care is likely to under-identify challenging behaviours because it is only people experiencing the most severe challenging behaviours who will have been assessed in primary care or seen by specialist services for people with intellectual disabilities. Relatedly, though compensated for using time-windows in the analyses, behaviours or health conditions may have been present for some time before being recognised or coded, meaning that recorded dares reflect documentation rather than onset.

Second, over 70% of the cohort had missing data on severity of intellectual disability. Although the sensitivity analyses performed to take account of these missing data did not result in a significant change in the findings (although the strength of associations was attenuated), improving the quality of data on severity of intellectual disability recorded in primary care settings, and in other datasets, would increase the rigour of future longitudinal studies investigating the health needs of people with intellectual disabilities.

We decided to include data up to 2019, to minimise potentially confounding effects of the COVID-19 pandemic on the assessment and diagnosis of physical health problems/sensory problems, mental health problems and challenging behaviours. People with intellectual disabilities were identified as being at high risk of severe outcomes of COVID-19 infection so were subject to more stringent restrictions around social contact and participation in community activities. Therefore, the COVID-19 lockdowns will have impacted on physical and mental health, and the incidence of challenging behaviours experienced by people with intellectual disabilities, so the use of data before 2020 will have increased the reliability of the findings reported here. However, we recognise that a future study using post COVID-19 data is needed to provide up-to-date findings that reflect the reduced quality of life and more limited access to effective health and social care that people with intellectual disabilities have experienced in the UK in the post-COVID period.^43 44^

Furthermore, while our design allows for inferences about temporality compared with cross-sectional studies, the observational nature of the data precludes definitive causal conclusions.

### Clinical and policy implications

The findings have significant implications for clinical practice, care planning, and public health policy. The findings reinforce clinical guidelines^33^ that recommend assessment for challenging behaviour should include screening for unrecognised physical health problems/sensory impairments. Our findings make it clear that a holistic assessment for challenging behaviour should consider, and indeed directly rule out, underlying physical health problems and sensory impairments before proceeding to psychological and systems intervention planning.^45^ Although the data used in this study were hosted in primary care where physical health problems/sensory impairments are diagnosed and managed, the responsibility for the assessment and management of challenging behaviours in the UK is mainly managed by secondary and tertiary health and social care services. Therefore, the holistic consideration of the complex relationships between challenging behaviours and physical health problems/sensory impairments can only be achieved by the provision of well-resourced, multidisciplinary, specialist intellectual disabilities or mental health services working effectively in partnership across primary, secondary and tertiary health and social care services. Streamlined communication is central to this partnership working, with acceptance that primary care services may be asked to assess and manage physical health problems/sensory impairments identified by specialist assessments of challenging behaviours.

### Future directions

While this study strengthens the evidence of a link between incident physical health problems/sensory impairments and the onset of challenging behaviours, we did not examine whether effective management of physical health problems/sensory impairments may lead to a reduction in the incidence of challenging behaviours or their ongoing severity or maintenance. Other evidence does suggest that treatment of physical health problems/sensory impairments can lead to reductions in challenging behaviour. For example, the review of case studies by Kennedy^39^ suggests that behavioural approaches can minimise the effect of sleep deprivation on challenging behaviours. However, there is a need for large scale evidence to examine the effectiveness and cost-effectiveness of the identification and treatment of physical health problems/sensory impairments as an intervention strategy in services.

A biopsychosocial approach to challenging behaviour also suggests that complex biological and psychological/social interactions are likely to occur in the maintenance of challenging behaviour which may then have implications for treatment beyond simply treating health conditions directly. For example, Bowring *et al*^12^ suggested that in the case of pain associated with the onset of challenging behaviours, the management of the pain, for example with analgesia, may in itself be reinforcing potentially leading to long-term maintenance of challenging behaviour. A further complexity when exploring the associations between challenging behaviours and physical health problems is introduced when psychotropic medication used to manage challenging behaviours potentially causes side effects of incontinence, constipation, disrupted sleep and occasionally seizures, and medications used to treat pain, epilepsy and incontinence can be associated with challenging behaviours and sleep changes. Therefore, future studies should aim to examine the possible complex interactions between physical health problems/sensory impairments, psychotropic and other medications and challenging behaviours, and the effectiveness of intervention approaches that address co-incident physical health problems/sensory impairments and challenging behaviours.

## Conclusions

Physical health problems/sensory impairments commonly act as biological vulnerability factors for the onset of incident challenging behaviours. Comprehensively screening for unrecognised physical health problems/sensory impairments should be a routine part of all assessments of incident challenging behaviours experienced by individuals with intellectual disabilities.

## Supplementary material

10.1136/bmjopen-2025-111117 online supplemental file 1

10.1136/bmjopen-2025-111117 online supplemental file 2

10.1136/bmjopen-2025-111117 online supplemental file 3

10.1136/bmjopen-2025-111117 online supplemental file 4

10.1136/bmjopen-2025-111117 online supplemental file 5

10.1136/bmjopen-2025-111117 online supplemental file 6

10.1136/bmjopen-2025-111117 online supplemental file 7

## Data Availability

Data may be obtained from a third party and are not publicly available.
