# Supplementary material for: Temporal associations between incident physical health problems/sensory impairments and challenging behaviours in people with intellectual disabilities: a population-based longitudinal cohort study of primary care in England
Source: BMJ Open. 2026 Jul 3;16(7):e111117. doi: 10.1136/bmjopen-2025-111117 (PMC13343113; doi:10.1136/bmjopen-2025-111117)
Supplement: online supplemental file 1 [file bmjopen-16-7-s001.docx]

**Table S1: Read Codes for challenging behaviours**

| **Read Code** | **Read Term** | **SNODMED ID** |
| --- | --- | --- |
| 1469 | H/O: behaviour problem | 161473006 |
| 2256 | O/E - agitated | 162721008 |
| 13HN000 | Theft | 248027001 |
| 13HN100 | Shoplifting | 248035003 |
| 13Z8000 | Social adjustment problem | 161153007 |
| 14OE.00 | At risk of harming others | 391155006 |
| 14X4.00 | On sex offenders register | 416081002 |
| 14X9.00 | Alleged perpetrator of physical abuse | 206381000000100 |
| 14XA.00 | Alleged perpetrator of sexual abuse | 206391000000103 |
| 14XB.00 | Alleged perpetrator of emotional abuse | 206401000000100 |
| 14XC.00 | Alleged perpetrator of domestic violence | 206411000000103 |
| 1B15.00 | Feeling irritable | 55929007 |
| 1B15.11 | Irritable - symptom | 55929007 |
| 1B16.00 | Agitated | 24199005 |
| 1B16.11 | Agitated - symptom | 24199005 |
| 1B1f.00 | Anger | 75408008 |
| 1B1I.00 | Crying, excessive | 162213003 |
| 1B1M.00 | Resents authority | 162219004 |
| 1B1O.00 | Restlessness | 162221009 |
| 1B1X.00 | Behavioural problem | 277843001 |
| 1BE1.00 | Problem situation | 276099005 |
| 1BN..00 | Wandering | 50239007 |
| 1BN0.00 | Wanders during the day | 310531007 |
| 1BN1.00 | Wanders at night | 310532000 |
| 1BN2.00 | Wanders during day and at night | 710560006 |
| 1P00.00 | Hyperactive behaviour | 44548000 |
| 1P02.00 | Disinhibited behaviour | 247973004 |
| 1P04.00 | C/O - akathisia | 416123004 |
| 1P10.00 | Clinging behaviour | 284605004 |
| 1P11.00 | Uncooperative behaviour | 248042003 |
| 1P12.00 | Suspiciousness and marked evasiveness | 274651006 |
| 1P2..00 | Habit | 90275003 |
| 1P3..00 | Compulsive behaviour | 12479006 |
| 1P30.00 | Compulsive uncontrollable drug taking | 416993008 |
| 1P31.00 | Compulsive drug taking | 416661002 |
| 1P4..00 | Vocal and verbal behaviour observations | 284592002 |
| 1P40.00 | Inappropriate shouting | 247984006 |
| 1P5..00 | Aggressive behaviour | 61372001 |
| 1P50.00 | Violent acts towards others | 401237008 |
| 1P51.00 | Physically abusive behaviour | 225480004 |
| 1P52.00 | Verbally abusive behaviour | 225478005 |
| 1P53.00 | Argumentative behaviour | 9182005 |
| 1P6..00 | Craving for drugs | 228438002 |
| 1P60.00 | Craves for drugs | 228438002 |
| 1P62.00 | Abnormal craving for drugs | 417298001 |
| 1P63.00 | Excessive craving for drugs | 417531009 |
| 1P64.00 | Irresistible craving for drugs | 417697003 |
| 1Q0..00 | Impulsive character | 286756000 |
| 225B.00 | O/E - angry | 162726003 |
| 287..00 | O/E - bizarre appearance | 163612006 |
| 288..11 | Uncooperative | 248042003 |
| 28C..00 | O/E - embarrassing behaviour | 268960007 |
| 28C..11 | O/E - irreverent behaviour | 275970006 |
| 28L..00 | O/E - impulsive behaviour | 417415004 |
| 3AB1.00 | Mildly abnormal behaviour | 165311008 |
| 3AB2.00 | Severely abnormal behaviour | 165312001 |
| 3AB3.00 | Change in behaviour | 315244000 |
| E213.00 | Explosive personality disorder | 231527003 |
| E213.11 | Aggressive character | 286798002 |
| E213.12 | Quarrelsome personality | 231527003 |
| E216.12 | Dependent personality | 84466009 |
| E216.13 | Labile personality | 191765005 |
| E224.00 | Exhibitionism | 58349009 |
| E224.11 | Flasher | 58349009 |
| E22y100 | Voyeurism | 63835008 |
| E273100 | Head-banging | 43954004 |
| E275111 | Compulsive eating disorder | 78004001 |
| E275200 | Pica | 14077003 |
| E275711 | Compulsive water drinking | 231511005 |
| E27z000 | Hair plucking | 17155009 |
| E27z300 | Masturbation | 17704007 |
| E27z400 | Nail biting | 37298006 |
| E292y00 | Adjustment reaction with mixed disturbance of emotion | 55668003 |
| E292z00 | Adjustment reaction with disturbance of other emotion NOS | 17226007 |
| E293.00 | Adjustment reaction with predominant disturbance of conduct | 84984002 |
| E293000 | Adjustment reaction with aggression | 192056005 |
| E293100 | Adjustment reaction with antisocial behaviour | 192057001 |
| E293200 | Adjustment reaction with destructiveness | 192058006 |
| E293z00 | Adjustment reaction with predominant disturbance conduct NOS | 84984002 |
| E294.00 | Adjustment reaction with mixed disturbance of emotion and conduct | 66381006 |
| E2C..00 | Conduct disorder | 430909002 |
| E2C..11 | Behavioural syndrome associated with physiological disturbance and physical factors | 231520001 |
| E2C0.00 | Aggressive unsocial conduct disorder | 192082001 |
| E2C0000 | Aggressive outburst | 192083006 |
| E2C0100 | Anger reaction | 192085004 |
| E2C0z00 | Aggressive unsocial conduct disorder NOS | 192082001 |
| E2C1.00 | Nonaggressive unsocial conduct disorder | 268661009 |
| E2C1100 | Solitary stealing | 268663007 |
| E2C1111 | Shop lifting | 248035003 |
| E2C1200 | Temper tantrum | 83943005 |
| E2C1z00 | Nonaggressive unsocial conduct disorder NOS | 268661009 |
| E2C2.00 | Conduct disorder - socialised | 386820009 |
| E2C2z00 | Socialised conduct disorder NOS | 386820009 |
| E2C3.00 | Impulse control disorder | 66347000 |
| E2C3000 | Impulse control disorder, unspecified | 66347000 |
| E2C3100 | Pathological gambling | 18085000 |
| E2C3200 | Kleptomania | 69361009 |
| E2C3300 | Pyromania | 600009 |
| E2C3400 | Intermittent explosive disorder | 40987004 |
| E2C3500 | Isolated explosive disorder | 192097003 |
| E2C3z00 | Impulse control disorder NOS | 66347000 |
| E2C4.00 | Mixed disturbance of conduct and emotion | 192099000 |
| E2C4000 | Neurotic delinquency | 192100008 |
| E2C4z00 | Mixed disturbance of conduct and emotion NOS | 192099000 |
| E2Cy.00 | Other conduct disturbances | 17961008 |
| E2Cy000 | Breath holding with temper | 90091006 |
| E2Cyz00 | Other conduct disturbances NOS | 17961008 |
| E2Cz.00 | Unspecified disturbance of conduct | 430909002 |
| E2Czz00 | Disturbance of conduct NOS | 430909002 |
| E2D1.13 | Taunting / tormenting | 60411006 |
| E2E2.00 | Hyperkinetic conduct disorder | 192132008 |
| Eu63.00 | Habit and impulse disorder | 66347000 |
| Eu63000 | [X]Pathological gambling | 18085000 |
| Eu63011 | Compulsive gambling | 18085000 |
| Eu63100 | [X]Pathological fire-setting | 600009 |
| Eu63200 | [X]Pathological stealing | 69361009 |
| Eu63300 | Trichotillomania | 17155009 |
| Eu63y00 | [X]Other habit and impulse disorders | 66347000 |
| Eu63z00 | [X]Habit and impulse disorder, unspecified | 66347000 |
| Eu65000 | [X]Fetishism | 59174009 |
| Eu65200 | [X]Exhibitionism | 58349009 |
| Eu65300 | [X]Voyeurism | 63835008 |
| Eu65y11 | [X]Frotteurism | 50299009 |
| Eu65z11 | [X]Sexual deviation NOS | 50299009 |
| Eu70100 | [X]Mld mental retard sig impairment behav req attent/treatmt | 86765009 |
| Eu70y00 | [X]Mild mental retardation, other impairments of behaviour | 86765009 |
| Eu71100 | [X]Mod mental retard sig impairment behav req attent/treatmt | 61152003 |
| Eu71y00 | [X]Mod retard oth behav impair | 61152003 |
| Eu72100 | [X]Sev mental retard sig impairment behav req attent/treatmt | 40700009 |
| Eu72y00 | [X]Severe mental retardation, other impairments of behaviour | 40700009 |
| Eu73100 | [X]Profound ment retard sig impairmnt behav req attent/treat | 31216003 |
| Eu73y00 | [X]Profound mental retardation, other impairments of behavr | 31216003 |
| Eu7y100 | [X]Oth mental retard sig impairment behav req attent/treatmt | 110359009 |
| Eu7yy00 | [X]Other mental retardation, other impairments of behaviour | 110359009 |
| Eu7z100 | [X]Unsp mentl retard sig impairment behav req attent/treatmt | 110359009 |
| Eu7zy00 | [X]Unspecified mental retardatn, other impairments of behav | 110359009 |
| Eu91.00 | [X]Conduct disorders | 430909002 |
| Eu91000 | [X]Conduct disorder confined to the family context | 231540008 |
| Eu91100 | Conduct disorder - unsocialised | 231541007 |
| Eu91111 | [X]Conduct disorder, solitary aggressive type | 231541007 |
| Eu91112 | [X]Unsocialised aggressive disorder | 231541007 |
| Eu91200 | [X]Socialized conduct disorder | 386820009 |
| Eu91211 | [X]Conduct disorder, group type | 386820009 |
| Eu91212 | [X]Group delinquency | 386820009 |
| E2C2300 | Group delinquency | 192092009 |
| Eu91213 | [X]Offences in the context of gang membership | 386820009 |
| Eu91214 | [X]Stealing in company of others | 386820009 |
| Eu91215 | [X]Truancy from school | 386820009 |
| Eu91300 | Oppositional defiant disorder | 18941000 |
| Eu91y00 | [X]Other conduct disorders | 17961008 |
| Eu91z00 | [X]Conduct disorder, unspecified | 430909002 |
| Eu91z11 | [X]Childhood behavioural disorder NOS | 430909002 |
| Eu91z12 | [X]Childhood conduct disorder NOS | 430909002 |
| Eu92.00 | [X]Mixed disorders of conduct and emotions | 5793009 |
| Eu92.11 | [X]Emotional behavioural problems | 5793009 |
| Eu92000 | [X]Depressive conduct disorder | 231542000 |
| Eu92y00 | [X]Other mixed disorders of conduct and emotions | 943171000000100 |
| Eu92y11 | [X]Conduct disorder associated with emotional disorder | 192099000 |
| Eu92y12 | [X]Conduct disorder associated with neurotic disorder | 192099000 |
| Eu92z00 | [X]Mixed disorder of conduct and emotions, unspecified | 192099000 |
| J08z000 | Cheek biting | 59901004 |
| J08z100 | Lip biting | 196575007 |
| R00z700 | Demoralisation | 45763006 |
| R00z800 | Irritability and anger | 274646000 |
| R00z900 | Hostile behaviour | 79351003 |
| R00zA00 | Physical aggression | 248004009 |
| R00zD00 | Restlessness and agitation | 274647009 |
| R2y3.11 | Self-neglect | 248054003 |
| Ry10.00 | Very low level of personal hygiene | 410428008 |
| Ry11.00 | Bizarre personal appearance | 80670004 |
| Ry12.00 | Strange and inexplicable behaviour | 274650007 |
| Ry13.00 | Behaviour showing increased motor activity | 28277004 |
| Ry14.00 | [D]Suspiciousness and marked evasiveness | 274651006 |
| Ry15.00 | Undue concern and preoccupation with stressful events | 274653009 |
| Ry18.00 | [D]Self neglect | 248054003 |
| U2...00 | Self-injurious behaviour | 248062006 |
| U2...11 | [X]Self inflicted injury | 248062006 |
| U2...12 | [X]Injury - self-inflicted | 248062006 |
| U2...13 | Suicide | 44301001 |
| U2...14 | [X]Attempted suicide | 82313006 |
| U2...15 | [X]Para-suicide | 48981002 |
| U20..00 | [X]Intentional self poisoning by and exposure to noxious substances | 461371000000105 |
| U20..11 | [X]Deliberate drug overdose / other poisoning | 461371000000105 |
| U200.00 | [X]Intentional self poisoning by and exposure to nonopioid analgesics | 446901000000100 |
| U200.11 | Paracetamol overdose | 295124009 |
| U200.12 | Ibuprofen overdose | 295250003 |
| U200.13 | [X]Overdose - aspirin | 446901000000100 |
| U200000 | Self-administered intentional poisoning by analgesic | 393341000000109 |
| U200100 | [X]Intentional self poisoning by and exposure to nonopioid analgesics, occurrence in residential institution | 474461000000102 |
| U200400 | [X]Intentional self poisoning by and exposure to nonopioid analgesics, occurrence on street and highway | 385711000000109 |
| U200500 | [X]Intentional self poisoning by and exposure to nonopioid analgesics, occurrence at trade and service area | 473621000000105 |
| U200600 | [X]Intentional self poisoning by and exposure to nonopioid analgesics, occurrence at industrial and construction area | 385731000000101 |
| U200y00 | [X]Intentional self poisoning by and exposure to nonopioid analgesics, occurrence at other specified place | 459381000000104 |
| U200z00 | [X]Intentional self poisoning by and exposure to nonopioid analgesics, occurrence at unspecified place | 459421000000108 |
| U201.00 | [X]Intentional self poisoning by and exposure to antiepileptics | 271982007 |
| U201000 | [X]Intentional self poisoning by and exposure to antiepileptics, occurrence at home | 42377002 |
| U201z00 | [X]Intentional self poisoning by and exposure to antiepileptics, occurrence at unspecified place | 42377002 |
| U202.00 | [X]Intentional self poisoning by and exposure to sedative hypnotics | 271982007 |
| U202.11 | Sedative overdose | 296015009 |
| U202.12 | Diazepam overdose | 296118008 |
| U202.13 | Overdose of temazepam | 296125001 |
| U202.15 | Nitrazepam overdose | 296070007 |
| U202.16 | Benzodiazepine overdose | 296053004 |
| U202.17 | Barbiturate overdose | 296036006 |
| U202.18 | Amobarbital overdose | 296037002 |
| U202000 | [X]Intentional self poisoning by and exposure to sedative hypnotics, occurrence at home | 85337000 |
| U202400 | [X]Intentional self poisoning by and exposure to sedative hypnotics, occurrence on street and highway | 85337000 |
| U202y00 | [X]Intentional self poisoning by and exposure to sedative hypnotics, occurrence at other specified place | 85337000 |
| U202z00 | [X]Intentional self poisoning by and exposure to sedative hypnotics, occurrence at unspecified place | 85337000 |
| U204.00 | [X]Intentional self poisoning by and exposure to psychotropic drugs | 271982007 |
| U204.11 | Overdose of antidepressant drug | 295830007 |
| U204.12 | Amitriptyline overdose | 295835002 |
| U204.13 | Selective serotonin re-uptake inhibitor overdose | 295918002 |
| U204000 | [X]Intentional self poisoning by and exposure to psychotropic drugs, occurrence at home | 271982007 |
| U204100 | [X]Intentional self poisoning by and exposure to psychotropic drugs, occurrence in residential institution | 61438005 |
| U204y00 | [X]Intentional self poisoning by and exposure to psychotropic drugs, occurrence at other specified place | 61438005 |
| U204z00 | [X]Intentional self poisoning by and exposure to psychotropic drugs, occurrence at unspecified place | 61438005 |
| U205.00 | Intentional narcotic poisoning | 290221007 |
| U205.11 | Heroin overdose | 295174006 |
| U205000 | [X]Intentional self poisoning by and exposure to narcotic drugs, occurrence at home | 433941000000106 |
| U205y00 | [X]Intentional self poisoning by and exposure to narcotic drugs, occurrence at other specified place | 458971000000107 |
| U205z00 | [X]Intentional self poisoning by and exposure to narcotic drugs, occurrence at unspecified place | 403681000000100 |
| U206.00 | [X]Intentional self poisoning by and exposure to hallucinogens | 271982007 |
| U206000 | [X]Intentional self poisoning by and exposure to hallucinogens, occurrence at home | 85975005 |
| U206400 | [X]Intentional self poisoning by and exposure to hallucinogens, occurrence on street and highway | 85975005 |
| U207.00 | [X]Intentional self poisoning by and exposure to other autonomic drugs | 271982007 |
| U207000 | [X]Intentional self poisoning by and exposure to other autonomic drugs, occurrence at home | 32835006 |
| U207z00 | [X]Intentional self poisoning by and exposure to other autonomic drugs, occurrence at unspecified place | 32835006 |
| U208.00 | [X]Int self poison/exposure to other/unspec drug/medicament | 271982007 |
| U208000 | [X]Int self poison/exposure to oth/unsp drug/medicam home | 271982007 |
| U208400 | [X]Intent self pois oth/unsp drug/medic in street/highway | 271982007 |
| U208y00 | [X]Int self poison oth/unsp drug/medic other spec place | 271982007 |
| U208z00 | [X]Intent self poison oth/unsp drug/medic unspecif place | 271982007 |
| U209.00 | [X]Intentional self poisoning by and exposure to alcohol | 271982007 |
| U209000 | [X]Intentional self poisoning by and exposure to alcohol, occurrence at home | 67426006 |
| U209y00 | [X]Intentional self poisoning by and exposure to alcohol, occurrence at other specified place | 67426006 |
| U209z00 | [X]Intentional self poisoning by and exposure to alcohol, occurrence at unspecified place | 67426006 |
| U20A.00 | [X]Intentional self poisoning by and exposure to organic solvents and halogenated hydrocarbons and their vapours | 271982007 |
| U20A.11 | [X]Self poisoning from glue solvent | 271982007 |
| U20A000 | [X]Intentional self poisoning by and exposure to organic solvents and halogenated hydrocarbons and their vapours, occurrence at home | 241769004 |
| U20A400 | [X]Intentional self poisoning by and exposure to organic solvents and halogenated hydrocarbons and their vapours, occurrence on street and highway | 241769004 |
| U20Az00 | [X]Intentional self poisoning by and exposure to organic solvents and halogenated hydrocarbons and their vapours, halogens, occurrence at unspecified place | 241769004 |
| U20B.00 | [X]Intent self poison/exposure to other gas/vapour | 271982007 |
| U20B.11 | [X]Self carbon monoxide poisoning | 271982007 |
| U20B000 | [X]Int self poison/exposure to other gas/vapour at home | 271982007 |
| U20B200 | [X]Int self poison other gas/vapour school/pub admin area | 271982007 |
| U20By00 | [X]Int self poison other gas/vapour other spec place | 271982007 |
| U20Bz00 | [X]Intent self poison other gas/vapour unspecif place | 271982007 |
| U20C.00 | [X]Intentional self poisoning by and exposure to pesticides | 461331000000108 |
| U20C.11 | [X]Self poisoning with weedkiller | 274912004 |
| U20C.12 | [X]Self poisoning with paraquat | 242837004 |
| U20C000 | [X]Intentional self poisoning by and exposure to pesticides, occurrence at home | 392701000000104 |
| U20Cy00 | [X]Intentional self poisoning by and exposure to pesticides, occurrence at other specified place | 445341000000107 |
| U20y.00 | [X]Intent self poison/exposure to unspecif chemical | 271982007 |
| U20y000 | [X]Int self poison/exposure to unspecif chemical at home | 271982007 |
| U20y200 | [X]Int self poison unspecif chemical school/pub admin area | 271982007 |
| U20yz00 | [X]Intent self poison unspecif chemical unspecif place | 271982007 |
| U21..00 | Self-destructive behavior | 248062006 |
| U210.00 | [X]Intent self harm by hanging strangulat/suffocat occ home | 434241000000100 |
| U211.00 | [X]Intent self harm by hangng strangult/suffoct resid instit | 473791000000101 |
| U212.00 | [X]Inten slf harm hang strang/suffc sch oth ins/pub adm area | 392751000000103 |
| U216.00 | [X]Intent self harm by hang strangl/suffc indust/constr area | 248062006 |
| U21y.00 | [X]Intent self harm by hangng strangul/suffoct oth spec plce | 248062006 |
| U21z.00 | [X]Intent self harm by hangng strangul/suffoct unspecif plce | 391821000000107 |
| U22..00 | [X]Intentional self harm by drowning and submersion | 446891000000101 |
| U220.00 | [X]Intentional self harm by drowning and submersion, occurrence at home | 458281000000101 |
| U221.00 | [X]Intentional self harm by drowning and submersion, occurrence in residential institution | 473731000000102 |
| U22y.00 | [X]Intentional self harm by drowning and submersion, occurrence at other specified place | 472681000000104 |
| U22z.00 | [X]Intentional self harm by drowning and submersion, occurrence at unspecified place | 415591000000101 |
| U24..00 | [X]Intentional self harm by rifle, shotgun and larger firearm discharge | 387051000000104 |
| U241.00 | [X]Intentional self harm by rifle, shotgun and larger firearm discharge, occurrence in residential institution | 393881000000109 |
| U242.00 | [X]Intentional self harm by rifle, shotgun and larger firearm discharge, occurrence at school, other institution and public administrative area | 446041000000101 |
| U25..00 | [X]Intentional self harm by other and unspecified firearm discharge | 403641000000108 |
| U250.00 | [X]Intentional self harm by other and unspecified firearm discharge, occurrence at home | 433281000000106 |
| U26..00 | [X]Intentional self harm by explosive material | 418420002 |
| U27..00 | [X]Intentional self harm by smoke, fire and flames | 418420002 |
| U270.00 | [X]Intentional self harm by smoke, fire and flames, occurrence at home | 418420002 |
| U274.00 | [X]Intentional self harm by smoke, fire and flames, occurrence on street and highway | 284744004 |
| U27z.00 | [X]Intentional self harm by smoke, fire and flames, occurrence at unspecified place | 284744004 |
| U28..00 | [X]Intentional self harm by steam, hot vapours and hot objects | 418420002 |
| U280.00 | [X]Intentional self harm by steam, hot vapours and hot objects, occurrence at home | 285142001 |
| U28z.00 | [X]Intentional self harm by steam, hot vapours and hot objects, occurrence at unspecified place | 285142001 |
| U29..00 | [X]Intentional self harm by sharp object | 248073004 |
| U290.00 | [X]Intentional self harm by sharp object, occurrence at home | 446551000000106 |
| U291.00 | [X]Intentional self harm by sharp object, occurrence in residential institution | 459811000000100 |
| U294.00 | [X]Intentional self harm by sharp object, occurrence on street and highway | 385451000000102 |
| U29y.00 | [X]Intentional self harm by sharp object, occurrence at other specified place | 433701000000106 |
| U29z.00 | [X]Intentional self harm by sharp object, occurrence at unspecified place | 445911000000107 |
| U2A..00 | [X]Intentional self harm by blunt object | 418420002 |
| U2A0.00 | [X]Intentional self harm by blunt object, occurrence at home | 284756003 |
| U2A1.00 | [X]Intentional self harm by blunt object, occurrence in residential institution | 284756003 |
| U2A3.00 | [X]Intentional self harm by blunt object, occurrence at sports and athletics area | 284756003 |
| U2B..00 | Attempted suicide - jumping from a high place | 287186005 |
| U2B0.00 | [X]Intentional self harm by jumping from a high place, occurrence at home | 287186005 |
| U2B4.00 | [X]Intentional self harm by jumping from a high place, occurrence on street and highway | 287186005 |
| U2B6.00 | [X]Intentional self harm by jumping from a high place, occurrence at industrial and construction area | 287186005 |
| U2By.00 | [X]Intentional self harm by jumping from a high place, occurrence at other specified place | 287186005 |
| U2Bz.00 | [X]Intentional self harm by jumping from a high place, occurrence at unspecified place | 392501000000107 |
| U2C..00 | [X]Intentional self harm by jumping or lying before moving object | 418420002 |
| U2C1.00 | [X]Intentional self harm by jumping or lying before moving object, occurrence in residential institution | 224948000 |
| U2C4.00 | [X]Intentional self harm by jumping or lying before moving object, occurrence on street and highway | 224948000 |
| U2Cy.00 | [X]Intentional self harm by jumping or lying before moving object, occurrence at other specified place | 224948000 |
| U2D..00 | [X]Intentional self harm by crashing of motor vehicle | 418420002 |
| U2D0.00 | [X]Intentional self harm by crashing of motor vehicle, occurrence at home | 418420002 |
| U2D4.00 | [X]Intentional self harm by crashing of motor vehicle, occurrence on street and highway | 418420002 |
| U2D6.00 | [X]Intentional self harm by crashing of motor vehicle, occurrence at industrial and construction area | 418420002 |
| U2E..00 | Self-mutilation | 130968006 |
| U2y..00 | Intentionally harming self | 418420002 |
| U2y0.00 | [X]Intentional self harm by other specified means, occurrence at home | 418420002 |
| U2y1.00 | [X]Intentional self harm by other specified means, occurrence in residential institution | 418420002 |
| U2y6.00 | [X]Intentional self harm by other specified means, occurrence at industrial and construction area | 418420002 |
| U2yz.00 | [X]Intentional self harm by other specified means, occurrence at unspecified place | 418420002 |
| U2z..00 | [X]Intentional self harm by unspecified means | 418420002 |
| U2z0.00 | [X]Intentional self harm by unspecified means, occurrence at home | 418420002 |
| U2z2.00 | [X]Intentional self harm by unspecified means, occurrence at school, other institution and public administrative area | 418420002 |
| U2zy.00 | [X]Intentional self harm by unspecified means, occurrence at other specified place | 418420002 |
| U2zz.00 | [X]Intentional self harm by unspecified means, occurrence at unspecified place | 418420002 |
| ZV40.11 | [V]Behavioural problems | 277843001 |
| ZV40300 | [V]Other behavioural problems | 277843001 |
| NA | SIB - Self-injurious behaviour | 248062006 |
| NA | SIB - Self-injurious behavior | 248062006 |
| NA | Deliberate self-harm | 248062006 |
| NA | Self-abusive behaviour | 248062006 |
| NA | Biting of oral mucosa - cheek | 59901004 |
| NA | Biting of oral mucosa - lip | 196575007 |
| TK6..00 | Suicide and selfinflicted injury by cutting and stabbing | 219150005 |
| TK60.00 | Suicide and selfinflicted injury by cutting | 219151009 |
| NA | Suicide or selfinflicted injury by cutting | 219151009 |
| TK6z.00 | Suicide and selfinflicted injury by cutting and stabbing NOS | 248062006 |
| NA | Injury of unknown intent by cutting instrument | 219343001 |
| NA | Suicide or selfinflicted injury by cutting and stabbing | 219150005 |
| TK1y.00 | Suicide and selfinflicted poisoning by other utility gas | 219125007 |
| TKz..00 | Suicide and selfinflicted injury NOS | 248062006 |
| TK05.00 | Intentional self poisoning | 271982007 |
| TK1..00 | Suicide + selfinflicted poisoning by gases in domestic use | 219125007 |
| TK2y.00 | Suicide + selfinflicted poisoning by other gases and vapours | 242839001 |
| TK01.00 | Suicide + selfinflicted poisoning by barbiturates | 296036006 |
| TKx7.00 | Suicide and selfinflicted injury caustic subst, excl poison | 403582001 |
| TK20.00 | Suicide + selfinflicted poisoning by motor veh exhaust gas | 219131005 |
| TK04.00 | Intentional poisoning by drug | 431307001 |
| TK08.00 | Suicide + selfinflicted poisoning by arsenic + its compounds | 219123000 |
| TK07.00 | Suicide + selfinflicted poisoning by corrosive/caustic subst | 219122005 |
| TK2..00 | Suicide + selfinflicted poisoning by other gases and vapours | 242839001 |
| TK30.00 | Attempted suicide - hanging | 287181000 |
| TK...00 | Suicide and selfinflicted injury | 248062006 |
| TK2z.00 | Suicide + selfinflicted poisoning by gases and vapours NOS | 242839001 |
| TKx0100 | Suicide + selfinflicted injury-lying before moving object | 418420002 |
| TK54.00 | Suicide and selfinflicted injury by other firearm | 287184008 |
| TKx0000 | Suicide + selfinflicted injury-jumping before moving object | 418420002 |
| TKx5.00 | Suicide and selfinflicted injury by crashing motor vehicle | 418420002 |
| TK4..00 | Suicide and selfinflicted injury by drowning | 287183002 |
| TKx0z00 | Suicide + selfinflicted inj-jump/lie before moving obj NOS | 418420002 |
| TK51.00 | Suicide and selfinflicted injury by shotgun | 287184008 |
| NA | Suicide and selfinflicted injury by drowning | 219141008 |
| TK71.00 | Suicide+selfinflicted injury-jump from oth manmade structure | 287186005 |
| TK5z.00 | Suicide and selfinflicted injury by firearms/explosives NOS | 287184008 |
| TK7z.00 | Suicide+selfinflicted injury-jump from high place NOS | 287186005 |
| TK70.00 | Suicide+selfinflicted injury-jump from residential premises | 287186005 |
| NA | Suicide or selfinflicted injury by stabbing | 219153007 |
| NA | Suicide and selfinflicted poisoning by analgesics, antipyretics and anti-rheumatics | 219108005 |
| TK50.00 | Suicide and selfinflicted injury by handgun | 287184008 |
| TK3y.00 | Suicide + selfinflicted inj oth mean hang/strangle/suffocate | 248062006 |
| TK31.00 | Suicide + selfinflicted injury by suffocation by plastic bag | 248062006 |
| TKxy.00 | Suicide and selfinflicted injury by other specified means | 248062006 |
| TKx..00 | Suicide and selfinflicted injury by other means | 248062006 |
| TKx0.00 | Suicide + selfinflicted injury-jump/lie before moving object | 418420002 |
| TKx2.00 | Suicide and selfinflicted injury by scald | 418420002 |
| TK02.00 | Suicide + selfinflicted poisoning by oth sedatives/hypnotics | 431307001 |
| NA | Suicide and selfinflicted injury | 269725004 |
| TK00.00 | Suicide + selfinflicted poisoning by analgesic/antipyretic | 219108005 |
| TK61.00 | Suicide and selfinflicted injury by stabbing | 219153007 |
| NA | Suicide and selfinflicted poisoning by gases in domestic use | 219125007 |
| TK72.00 | Suicide+selfinflicted injury-jump from natural sites | 287186005 |
| TK21.00 | Suicide and selfinflicted poisoning by other carbon monoxide | 271982007 |
| TKxz.00 | Suicide and selfinflicted injury by other means NOS | 248062006 |
| TK5..00 | Suicide and selfinflicted injury by firearms and explosives | 287184008 |
| TK52.00 | Suicide and selfinflicted injury by hunting rifle | 287184008 |
| TK10.00 | Suicide and selfinflicted poisoning by gas distributed by pipeline | 219126008 |
| NA | Suicide and selfinflicted injury by suffocation by plastic bag | 269726003 |
| NA | Suicide or selfinflicted injury by firearms and explosives | 219142001 |
| TK0z.00 | Suicide + selfinflicted poisoning by solid/liquid subst NOS | 242836008 |
| TK1z.00 | Suicide + selfinflicted poisoning by domestic gases NOS | 219125007 |
| TKx6.00 | Suicide and selfinflicted injury by crashing of aircraft | 248062006 |
| TK3..00 | Suicide + selfinflicted injury by hang/strangulate/suffocate | 248062006 |
| TK3z.00 | Suicide + selfinflicted inj by hang/strangle/suffocate NOS | 248062006 |
| TK0..00 | Self poisoning by non-drug solid or liquid agents | 242836008 |
| TKx3.00 | Suicide and selfinflicted injury by extremes of cold | 418420002 |
| TK03.00 | Suicide + selfinflicted poisoning tranquilliser/psychotropic | 431307001 |
| TKx4.00 | Suicide and selfinflicted injury by electrocution | 219168008 |
| TK11.00 | Suicide and selfinflicted poisoning by liquefied petroleum gas distributed in mobile containers | 219127004 |
| TK06.00 | Suicide + selfinflicted poisoning by agricultural chemical | 274912004 |
| TKy..00 | Late effects of selfinflicted injury | 219174008 |
| TK7..00 | Suicide and selfinflicted injury by jumping from high place | 287186005 |
| TKx1.00 | Suicide and selfinflicted injury by burns or fire | 418420002 |
| TK55.00 | Suicide and selfinflicted injury by explosives | 418420002 |
| TK53.00 | Suicide and selfinflicted injury by military firearms | 287184008 |
| TK60111 | Slashed wrists self inflicted | 219152002 |
| U48..00 | [X]Contact with steam, hot vapours and hot objects, undetermined intent | 419048002 |
| TK4..98 | Suicide - drowning | 219141008 |
| U13z700 | [X]Unspecified drowning and submersion, occurrence on farm | 409544000 |
| U13z000 | [X]Unspecified drowning and submersion, occurrence at home | 409544000 |
| U13z200 | [X]Unspec drowning+submer occ at sch oth inst/pub admin area | 409544000 |
| U13z100 | [X]Unspecif drowning+submersion occurrn in resid instit'n | 409544000 |
| U13zy00 | [X]Unspecif drowning+submersion occurrn at oth specif place | 409544000 |
| U13z300 | [X]Unspecif drowning+submersion occurrn sport/athletic area | 409544000 |
| U13zz00 | [X]Unspecif drowning+submersion occurrn at unspecif place | 409544000 |
| U13z600 | [X]Unspecif drowning+submersion occurrn indust/constr area | 409544000 |
| U13z500 | [X]Unspecif drowning+submersion occurrn trade/service area | 409544000 |
| TK4..99 | Attempt suicide - drowning | 287183002 |
| NA | [X]Intentional self harm by drowning and submersion, occurrence at unspecified place | 415591000000101 |
| NA | [X]Intentional self harm by drowning and submersion, occurrence at other specified place | 472681000000104 |
| NA | [X]Intentional self harm by drowning and submersion | 446891000000101 |
| U227.00 | [X]Intentional self harm by drowning and submersion, occurrence on farm | 392031000000107 |
| U224.00 | [X]Intentional self harm by drowning and submersion, occurrence on street and highway | 457331000000107 |
| NA | Attempted suicide - drowning | 287183002 |
| NA | [X]Intentional self harm by drowning and submersion, occurrence at school, other institution and public administrative area | 457321000000105 |
| NA | [X]Intentional self harm by drowning and submersion, occurrence in residential institution | 473731000000102 |
| NA | [X]Intentional self harm by drowning and submersion, occurrence at home | 458281000000101 |
| NA | [X]Intentional self harm by drowning and submersion, occurrence at trade and service area | 392011000000104 |
| NA | [X]Intentional self harm by drowning and submersion, occurrence on farm | 392031000000107 |
| U13z.00 | [X]Unspecified drowning and submersion | 409544000 |
| NA | [X]Intentional self harm by drowning and submersion, occurrence on street and highway | 457331000000107 |
| NA | [X]Intentional self harm by drowning and submersion, occurrence at sports and athletics area | 385231000000108 |
| NA | [X]Intentional self harm by drowning and submersion, occurrence at industrial and construction area | 392021000000105 |
| U2Az.00 | [X]Intentional self harm by blunt object, occurrence at unspecified place | 284756003 |
| U2z7.00 | [X]Intentional self harm by unspecified means, occurrence on farm | 418420002 |
| NA | [X]Intentional self harm by other and unspecified firearm discharge | 403641000000108 |
| NA | [X]Intentional self harm by sharp object, occurrence on street and highway | 385451000000102 |
| NA | [X]Intentional self harm by handgun discharge, occurrence at trade and service area | 458381000000105 |
| NA | [X]Intentional self harm by sharp object, occurrence on farm | 385501000000104 |
| NA | [X]Intentional self harm by rifle, shotgun and larger firearm discharge | 387051000000104 |
| NA | [X]Intentional self harm by other and unspecified firearm discharge, occurrence at unspecified place | 445901000000105 |
| NA | [X]Intentional self harm by handgun discharge, occurrence at sports and athletics area | 405061000000100 |
| NA | [X]Intentional self harm by handgun discharge, occurrence on street and highway | 393111000000106 |
| NA | [X]Intentional self harm by sharp object, occurrence in residential institution | 459811000000100 |
| NA | [X]Intentional self harm by sharp object, occurrence at sports and athletics area | 385441000000100 |
| NA | [X]Intentional self harm by rifle, shotgun and larger firearm discharge, occurrence at school, other institution and public administrative area | 446041000000101 |
| NA | [X]Intentional self harm by other and unspecified firearm discharge, occurrence at industrial and construction area | 457721000000106 |
| NA | [X]Intentional self harm by sharp object, occurrence at industrial and construction area | 385491000000105 |
| NA | [X]Intentional self harm by sharp object, occurrence at trade and service area | 385461000000104 |
| NA | [X]Intentional self harm by sharp object, occurrence at school, other institution and public administrative area | 459821000000106 |
| NA | [X]Intentional self harm by other and unspecified firearm discharge, occurrence on farm | 385481000000108 |
| NA | [X]Intentional self harm by other and unspecified firearm discharge, occurrence at other specified place | 445891000000109 |
| NA | [X]Intentional self harm by hanging, strangulation and suffocation, occurrence at school, other institution and public administrative area | 392751000000103 |
| NA | [X]Intentional self harm by handgun discharge, occurrence at unspecified place | 393841000000101 |
| NA | [X]Intentional self harm by rifle, shotgun and larger firearm discharge, occurrence at other specified place | 432991000000105 |
| NA | [X]Intentional self harm by rifle, shotgun and larger firearm discharge, occurrence in residential institution | 393881000000109 |
| NA | [X]Intentional self harm by hanging, strangulation and suffocation, occurrence at unspecified place | 391821000000107 |
| NA | [X]Intentional self harm by other and unspecified firearm discharge, occurrence at school, other institution and public administrative area | 403671000000102 |
| NA | [X]Intentional self harm by rifle, shotgun and larger firearm discharge, occurrence on street and highway | 446251000000108 |
| NA | [X]Intentional self harm by rifle, shotgun and larger firearm discharge, occurrence at home | 393871000000107 |
| U297.00 | [X]Intentional self harm by sharp object, occurrence on farm | 385501000000104 |
| NA | [X]Intentional self harm by handgun discharge, occurrence at home | 420511000000107 |
| NA | [X]Intentional self harm by jumping from a high place, occurrence at unspecified place | 392501000000107 |
| NA | [X]Intentional self harm by rifle, shotgun and larger firearm discharge, occurrence at sports and athletics area | 421331000000103 |
| NA | [X]Intentional self harm by other and unspecified firearm discharge, occurrence in residential institution | 472751000000101 |
| NA | [X]Intentional self harm by handgun discharge, occurrence at school, other institution and public administrative area | 405051000000103 |
| NA | [X]Intentional self harm by rifle, shotgun and larger firearm discharge, occurrence at trade and service area | 473371000000101 |
| NA | [X]Intentional self harm by other and unspecified firearm discharge, occurrence on street and highway | 404831000000101 |
| NA | [X]Intentional self harm by hanging, strangulation and suffocation, occurrence in residential institution | 473791000000101 |
| NA | [X]Intentional self harm by rifle, shotgun and larger firearm discharge, occurrence on farm | 404741000000103 |
| NA | [X]Intentional self harm by other and unspecified firearm discharge, occurrence at home | 433281000000106 |
| NA | [X]Intentional self harm by other and unspecified firearm discharge, occurrence at trade and service area | 458531000000103 |
| NA | [X]Intentional self harm by sharp object, occurrence at other specified place | 433701000000106 |
| NA | [X]Intentional self harm by handgun discharge, occurrence at industrial and construction area | 458391000000107 |
| NA | [X]Intentional self harm by handgun discharge | 472701000000102 |
| U2A7.00 | [X]Intentional self harm by blunt object, occurrence on farm | 284756003 |
| NA | [X]Intentional self harm by sharp object, occurrence at unspecified place | 445911000000107 |
| NA | [X]Intentional self harm by handgun discharge, occurrence at other specified place | 422691000000103 |
| NA | [X]Intentional self harm by handgun discharge, occurrence on farm | 434261000000104 |
| NA | [X]Intentional self harm by other and unspecified firearm discharge, occurrence at sports and athletics area | 445351000000105 |
| NA | [X]Intentional self harm by sharp object, occurrence at home | 446551000000106 |
| NA | [X]Intentional self harm by rifle, shotgun and larger firearm discharge, occurrence at industrial and construction area | 473381000000104 |
| NA | [X]Intentional self harm by rifle, shotgun and larger firearm discharge, occurrence at unspecified place | 445021000000102 |
| NA | [X]Intentional self harm by handgun discharge, occurrence in residential institution | 391801000000103 |
| U23..00 | [X]Intentional self harm by handgun discharge | 472701000000102 |
| NA | [X]Intentional self harm by hanging, strangulation and suffocation, occurrence at home | 434241000000100 |
| U2A2.00 | [X]Intentional self harm by blunt object, occurrence at school, other institution and public administrative area | 284756003 |
| U2D7.00 | [X]Intentional self harm by crashing of motor vehicle, occurrence on farm | 418420002 |
| U265.00 | [X]Intentional self harm by explosive material, occurrence at trade and service area | 219340003 |
| U284.00 | [X]Intentional self harm by steam, hot vapours and hot objects, occurrence on street and highway | 285142001 |
| U2A5.00 | [X]Intentional self harm by blunt object, occurrence at trade and service area | 284756003 |
| U2D3.00 | [X]Intentional self harm by crashing of motor vehicle, occurrence at sports and athletics area | 418420002 |
| U2D1.00 | [X]Intentional self harm by crashing of motor vehicle, occurrence in residential institution | 418420002 |
| U266.00 | [X]Intentional self harm by explosive material, occurrence at industrial and construction area | 219340003 |
| U276.00 | [X]Intentional self harm by smoke, fire and flames, occurrence at industrial and construction area | 284744004 |
| U26z.00 | [X]Intentional self harm by explosive material, occurrence at unspecified place | 219340003 |
| U275.00 | [X]Intentional self harm by smoke, fire and flames, occurrence at trade and service area | 284744004 |
| U263.00 | [X]Intentional self harm by explosive material, occurrence at sports and athletics area | 219340003 |
| U287.00 | [X]Intentional self harm by steam, hot vapours and hot objects, occurrence on farm | 285142001 |
| U28y.00 | [X]Intentional self harm by steam, hot vapours and hot objects, occurrence at other specified place | 285142001 |
| U2A6.00 | [X]Intentional self harm by blunt object, occurrence at industrial and construction area | 284756003 |
| U2C0.00 | [X]Intentional self harm by jumping or lying before moving object, occurrence at home | 224948000 |
| U2C7.00 | [X]Intentional self harm by jumping or lying before moving object, occurrence on farm | 224948000 |
| U273.00 | [X]Intentional self harm by smoke, fire and flames, occurrence at sports and athletics area | 284744004 |
| U2D5.00 | [X]Intentional self harm by crashing of motor vehicle, occurrence at trade and service area | 418420002 |
| U281.00 | [X]Intentional self harm by steam, hot vapours and hot objects, occurrence in residential institution | 285142001 |
| U2B1.00 | [X]Intentional self harm by jumping from a high place, occurrence in residential institution | 287186005 |
| U2B5.00 | [X]Intentional self harm by jumping from a high place, occurrence at trade and service area | 287186005 |
| U2y4.00 | [X]Intentional self harm by other specified means, occurrence on street and highway | 418420002 |
| U2yy.00 | [X]Intentional self harm by other specified means, occurrence at other specified place | 418420002 |
| U2B3.00 | [X]Intentional self harm by jumping from a high place, occurrence at sports and athletics area | 287186005 |
| U2z1.00 | [X]Intentional self harm by unspecified means, occurrence in residential institution | 418420002 |
| U2z5.00 | [X]Intentional self harm by unspecified means, occurrence at trade and service area | 418420002 |
| U225.00 | [X]Intentional self harm by drowning and submersion, occurrence at trade and service area | 392011000000104 |
| U231.00 | [X]Intentional self harm by handgun discharge, occurrence in residential institution | 391801000000103 |
| U240.00 | [X]Intentional self harm by rifle, shotgun and larger firearm discharge, occurrence at home | 393871000000107 |
| U233.00 | [X]Intentional self harm by handgun discharge, occurrence at sports and athletics area | 405061000000100 |
| U25z.00 | [X]Intentional self harm by other and unspecified firearm discharge, occurrence at unspecified place | 445901000000105 |
| U226.00 | [X]Intentional self harm by drowning and submersion, occurrence at industrial and construction area | 392021000000105 |
| U251.00 | [X]Intentional self harm by other and unspecified firearm discharge, occurrence in residential institution | 472751000000101 |
| U254.00 | [X]Intentional self harm by other and unspecified firearm discharge, occurrence on street and highway | 404831000000101 |
| U223.00 | [X]Intentional self harm by drowning and submersion, occurrence at sports and athletics area | 385231000000108 |
| U234.00 | [X]Intentional self harm by handgun discharge, occurrence on street and highway | 393111000000106 |
| U295.00 | [X]Intentional self harm by sharp object, occurrence at trade and service area | 385461000000104 |
| U296.00 | [X]Intentional self harm by sharp object, occurrence at industrial and construction area | 385491000000105 |
| U215.00 | [X]Intent self harm by hang strangl/suffc trade/service area | 248062006 |
| U23z.00 | [X]Intentional self harm by handgun discharge, occurrence at unspecified place | 393841000000101 |
| U232.00 | [X]Intentional self harm by handgun discharge, occurrence at school, other institution and public administrative area | 405051000000103 |
| U26y.00 | [X]Intentional self harm by explosive material, occurrence at other specified place | 219340003 |
| U292.00 | [X]Intentional self harm by sharp object, occurrence at school, other institution and public administrative area | 459821000000106 |
| U217.00 | [X]Intent self harm by hanging strangulat/suffocat occ farm | 248062006 |
| U222.00 | [X]Intentional self harm by drowning and submersion, occurrence at school, other institution and public administrative area | 457321000000105 |
| U214.00 | [X]Intent self harm by hangng strangult/suffoct street/h'way | 248062006 |
| U255.00 | [X]Intentional self harm by other and unspecified firearm discharge, occurrence at trade and service area | 458531000000103 |
| U2Dy.00 | [X]Intentional self harm by crashing of motor vehicle, occurrence at other specified place | 418420002 |
| U271.00 | [X]Intentional self harm by smoke, fire and flames, occurrence in residential institution | 284744004 |
| U236.00 | [X]Intentional self harm by handgun discharge, occurrence at industrial and construction area | 458391000000107 |
| U235.00 | [X]Intentional self harm by handgun discharge, occurrence at trade and service area | 458381000000105 |
| U213.00 | [X]Intent self harm by hang strangl/suffc sport/athlet area | 248062006 |
| U2B7.00 | [X]Intentional self harm by jumping from a high place, occurrence on farm | 287186005 |
| U2y3.00 | [X]Intentional self harm by other specified means, occurrence at sports and athletics area | 418420002 |
| U27y.00 | [X]Intentional self harm by smoke, fire and flames, occurrence at other specified place | 284744004 |
| U293.00 | [X]Intentional self harm by sharp object, occurrence at sports and athletics area | 385441000000100 |
| U2y5.00 | [X]Intentional self harm by other specified means, occurrence at trade and service area | 418420002 |
| U2z4.00 | [X]Intentional self harm by unspecified means, occurrence on street and highway | 418420002 |
| U262.00 | [X]Intentional self harm by explosive material, occurrence at school, other institution and public administrative area | 219340003 |
| U25y.00 | [X]Intentional self harm by other and unspecified firearm discharge, occurrence at other specified place | 445891000000109 |
| U257.00 | [X]Intentional self harm by other and unspecified firearm discharge, occurrence on farm | 385481000000108 |
| U247.00 | [X]Intentional self harm by rifle, shotgun and larger firearm discharge, occurrence on farm | 404741000000103 |
| U23y.00 | [X]Intentional self harm by handgun discharge, occurrence at other specified place | 422691000000103 |
| U2y2.00 | [X]Intentional self harm by other specified means, occurrence at school, other institution and public administrative area | 418420002 |
| U2z3.00 | [X]Intentional self harm by unspecified means, occurrence at sports and athletics area | 418420002 |
| U2Dz.00 | [X]Intentional self harm by crashing of motor vehicle, occurrence at unspecified place | 418420002 |
| U2z6.00 | [X]Intentional self harm by unspecified means, occurrence at industrial and construction area | 418420002 |
| U272.00 | [X]Intentional self harm by smoke, fire and flames, occurrence at school, other institution and public administrative area | 284744004 |
| NA | Head banging | 43954004 |
| TK30.99 | Attempt suicide - hanging | 287181000 |
| NA | Patient found hanging | 241121000000102 |
| NA | Suicide or attempted suicide by hanging | 219137009 |
| TK30.98 | Suicide - hanging | 219137009 |
| NA | Attempted suicide - jumping from a high place | 287186005 |
| NA | Suicide or self injury by jumping from a height | 219155000 |
| U4B..00 | [X]Falling, jumping or pushed from a high place, undetermined intent | 429482004 |
| U120100 | [X]Hit, struck, kicked, twisted, bitten or scratched by another person, occurrence in residential institution | 428200002 |
| U120000 | [X]Hit, struck, kicked, twisted, bitten or scratched by another person, occurrence at home | 428200002 |
| U120.00 | [X]Hit, struck, kicked, twisted, bitten or scratched by another person | 439022003 |
| U120700 | [X]Hit, struck, kicked, twisted, bitten or scratched by another person, occurrence on farm | 428200002 |
| NA | Hanging, strangulation or suffocation of unknown intent | 219328003 |
| NA | Attempted suicide - suffocation | 287182007 |
| U41..00 | [X]Hanging, strangulation and suffocation undetermined intent | 219328003 |
| NA | Suicide or attempted suicide by suffocation with plastic bag | 269726003 |
| NA | Self-scalding | 285142001 |
| TK5..98 | Suicide - firearms | 219142001 |
| 146A.00 | H/O: attempted suicide | 161474000 |
| 1BD7.00 | Low suicide risk | 394687007 |
| 1BD5.00 | High suicide risk | 394685004 |
| 388s.00 | Pierce suicide intent scale score | 428442002 |
| NA | Attempts suicide/homicide | 981161000006106 |
| NA | Pierce suicide intent scale | 1583961000006100 |
| NA | FH: Attempted suicide | 1807531000006100 |
| NA | Recent suicide attempt | 1821291000006100 |
| NA | Further suicide/self-harm risk assessment required | 1809311000006100 |
| NA | Threatening suicide | 41501003 |
| NA | MHIP comprehensive assessment - high risk of suicide | 2001351000006100 |
| NA | MHIP comprehensive assessment - current suicide plans | 2001011000006100 |
| NA | MHIP behavioural history - previous aborted suicide attempts | 2000301000006100 |
| NA | MHIP comprehensive assessment - low risk of suicide | 2001321000006100 |
| 9j2..00 | Initiation of suicide risk management document | 827321000000103 |
| 1S8..00 | No apparent risk of suicide | 826061000000105 |
| NA | MHIP comprehensive assessment - medium risk of suicide | 2001341000006100 |
| NA | MHIP - specific/serious/feasible suicide plans | 2001021000006100 |
| NA | MHIP - suicide plans prepared | 2001061000006100 |
| NA | MHIP comprehensive assessment - risk of suicide | 2001311000006100 |
| 8G6Z.00 | Anti-suicide psychotherapy NOS | 183401008 |
| TK...17 | Parasuicide | 48981002 |
| TK6..99 | Attempt suicide - cut/stab | 287185009 |
| NA | Family history of suicide | 160333008 |
| TK20.99 | Attempt suicide - car exhaust | 269808005 |
| TK31.99 | Attempt suicide - suffocate | 287182007 |
| NA | Planning suicide | 247650009 |
| NA | Attempt suicide - domestic gas | 288311002 |
| NA | Attempted suicide - firearms | 287184008 |
| TK05.99 | Attempt suicide -drug overdose | 681111000000102 |
| TKz..99 | Attempt suicide - NOS | 684281000000100 |
| TK...98 | Attempt suicide - NOS | 82313006 |
| TK7..99 | Attempt suicide-jump from high | 287186005 |
| NA | Attempt suicide - car exhaust | 269808005 |
| NA | Attempted suicide - cut/stab | 287185009 |
| NA | MHIP comprehensive assessment - no foreseen risk of suicide | 2001331000006100 |
| NA | At risk for suicide | 225444004 |
| TK5..99 | Attempt suicide - firearms | 287184008 |
| 8G61.00 | Potential suicide care | 183402001 |
| NA | MHIP - confidence to complete suicide plans | 2001101000006100 |
| NA | MHIP - intention to act on suicide plans | 2001461000006100 |
| NA | History of attempted suicide | 161474000 |
| TK1..99 | Attempt suicide - domestic gas | 288311002 |
| NA | PHQ9 score - thoughts of suicide or self harm | 1008751000006100 |
| 8G6..00 | Anti-suicide psychotherapy | 183401008 |
| NA | Non-fatal suicide behaviour | 1809461000006100 |
| 1BD6.00 | Moderate suicide risk | 394686003 |
| TK...15 | Suicide attempt | 82313006 |
| NA | MHIP - resources to act on suicide plans | 2001071000006100 |
| NA | Self mutilation | 130968006 |
| 8Cu2.00 | Drilling toenails of right foot | 932181000000108 |
| 8Cu1.00 | Drilling toenails of left foot | 932161000000104 |
| NA | [X]Intentional self poisoning by and exposure to noxious substances | 461371000000105 |
| NA | Throwing self in front of vehicle | 224948000 |
| NA | Jumping in front of vehicle | 224948000 |
| 1BV1.00 | Unable to comprehend concept of danger | 395663002 |
| NA | Difficulty understanding concept of danger | 395664008 |
| NA | Unable to understand concept of danger | 395663002 |
| 1BV2.00 | Difficulty comprehending concept of danger | 395664008 |
