# Supplementary material for: Temporal associations between incident physical health problems/sensory impairments and challenging behaviours in people with intellectual disabilities: a population-based longitudinal cohort study of primary care in England
Source: BMJ Open. 2026 Jul 3;16(7):e111117. doi: 10.1136/bmjopen-2025-111117 (PMC13343113; doi:10.1136/bmjopen-2025-111117)
Supplement: online supplemental file 2 [file bmjopen-16-7-s002.pdf]

General information

Protocol reference Id
20_000270

Study title
Association of physical conditions with challenging behaviours in people with intellectual disabilities: a longitudinal cohort study of a national sample of primary-care patients in England using the Clinical Practice Research Datalink

Research Area

Disease Epidemiology

Does this protocol describe an observational study using purely CPRD data?
Yes

Does this protocol involve requesting any additional information from GPs, or contact with patients?
No

3

Access to data

Sponsor
University of Glasgow

Funding source for the study
Is the funding source for the study the same as Chief Investigator's affiliation?
Yes
Funding source for the study
University of Glasgow

Institution conducting the research
Is the institution conducting the research the same as Chief Investigator's affiliation?
Yes
Institution conducting the research
University of Glasgow

Method to access the data
Indicate the method that will be used to access the data
Institutional multi-study licence
Is the institution the same as Chief Investigator's affiliation?
Yes
Institution name
University of Glasgow

Extraction by CPRD
Will the dataset be extracted by CPRD
No

Multiple data delivery
This study requires multiple data extractions over its lifespan
No

Data processors

**Data processor is**	Same as the chief investigator's affiliation
**Processing**	Yes
**Accessing**	Yes
**Storing**	Yes
**Processing area**	UK

4

Information on data

Primary care data

CPRD GOLD

CPRD Aurum

Do you require data linkages

Yes

Patient level data

HES Admitted Patient Care

Mental Health Services Data Set (MHSDS)

NCRAS data

Covid 19 linkages

Area level data

Do you require area level data?

Yes

Practice level (UK)

Practice Level Index of Multiple Deprivation

Patient level (England only)

Patient Level Index of Multiple Deprivation

Withheld concepts

Are withheld concepts required?
No

Linkage to a dataset not listed

Are you requesting a linkage to a dataset not listed?
No

Patient data privacy

Does any person named in this application already have access to any of these data in a patient identifiable form, or associated with an identifiable patient index?
No

5

Protocol information

Lay Summary

Previous studies have shown that people with intellectual disabilities commonly engage in challenging behaviours such as physically, verbally or sexually aggressive, destructive or self-injurious behaviour, but prevalence rates vary significantly between studies. Association of challenging behaviours with behavioural, psychiatric and psychosocial factors has been established previously, but physical conditions such as pain, sleep problems, constipation, epilepsy, urinary/bowel incontinence and sensory impairments have been less studied and existing evidence in mostly of low quality and inconclusive. In this study, we will investigate the incidence of challenging behaviours in a large nationwide population of primary care patients with intellectual disabilities. We will also examine the association between challenging behaviours and physical conditions in this population, including exploring how different health (e.g. severity of intellectual disabilities, co-occurring autism/genetic syndromes/mental comorbidities), demographic (e.g. age, gender, ethnicity) and socioeconomic (e.g. Practice/Patient Level Index of Multiple Deprivation) factors may affect this relationship. The results of this study will help us understand whether presence of challenging behaviours in patients with intellectual disabilities is affected by their physical health and how other factors may further impact this association.

Technical Summary

Challenging behaviours can include physically, verbally or sexually aggressive, destructive or self-injurious behaviour. The estimated prevalence of challenging behaviour problems in intellectual disabilities in large population studies shows considerable variation between 4–22%, with multiple factors likely to underlie challenging behaviours in this population. Risk factors associated with challenging behaviours in people with intellectual disabilities include male gender, severe/profound level of intellectual disabilities, co-occurring autism and deficits in receptive and expressive communication. An association with behavioural, psychiatric and psychosocial factors has also been established, but physical conditions such as pain, sleep problems, constipation, epilepsy, urinary/bowel incontinence and sensory impairments have been less studied and existing evidence is mostly of low quality and inconclusive. In this study, we will investigate the incidence of challenging behaviours in a large nationwide population of primary care patients with intellectual disabilities in England. Using stratified Cox proportional hazard models, we will also examine the association between challenging behaviours and physical conditions (i.e. pain, sleep problems, constipation, epilepsy, urinary/bowel incontinence and sensory impairments) in this population, including exploring how different health (e.g. severity of intellectual disabilities, co-occurring autism/genetic syndromes/mental comorbidities), demographic (e.g. age, gender, ethnicity) and socioeconomic (e.g. Practice/Patient Level Index of Multiple Deprivation) factors may affect this relationship.

Outcomes to be measured

Primary outcome: challenging behaviours as defined through READ codes in Appendix 1

Moderator: record of co-occurring physical conditions (i.e. pain, sleep problems, constipation, epilepsy, urinary/bowel incontinence and sensory impairments) as defined through READ codes

Mediators: severity of intellectual disabilities, co-occurring autism/genetic syndromes/mental comorbidities as defined through READ codes in Appendix 1

Confounding covariates: demographic (age, gender, ethnicity) and socioeconomic (Practice/Patient Level Index of Multiple Deprivation) factors

Objectives, specific aims & rationale

The aim of this project will be firstly to investigate longitudinal trends in the incidence of challenging behaviours in a nationwide population of primary care patients with intellectual disabilities, using data from the Clinical Practice Research Datalink. We will also investigate if there is an association between physical conditions and challenging behaviours in patients with intellectual disabilities and what the nature and direction of this association may be.

The project aims to answer the following research questions:
1. What is the 1-, 5- and 10-year incidence of challenging behaviours in a nationwide population of primary care patients with intellectual disabilities?
2. Do physical conditions (i.e. pain, sleep problems, constipation, epilepsy, urinary/bowel incontinence and sensory impairments) increase/decrease the incidence of challenging behaviours in primary care patients with intellectual disabilities?
3. To what extent do health factors (i.e. severity of intellectual disabilities, co-occurring autism/genetic syndromes/mental comorbidities) affect the incidence of challenging behaviours in primary care patients with intellectual disabilities?
4. Is the relationship between the presence of physical conditions and challenging behaviours in patients with intellectual disabilities accounted for by demographic (e.g. age, gender, ethnicity) and socioeconomic (e.g. Practice/Patient Level Index of Multiple Deprivation) factors?

Study background

Challenging behaviours can include physically, verbally or sexually aggressive, destructive or self-injurious behaviour (van den Akker et al. 2020). The estimated prevalence of challenging behaviour problems in intellectual disabilities in large population studies shows considerable variation between 4–22% (Bowring et al. 2019), with multiple factors likely to underlie challenging behaviours in this population.

Results of a meta-analysis of risk factors associated with challenging behaviours in people with intellectual disabilities indicate that men with intellectual disabilities are significantly more likely to show aggression than women, and that individuals with severe/profound intellectual disabilities are significantly more likely to show self-injury and stereotypy than individuals with mild/moderate intellectual disabilities (McClintock et al. 2003). Individuals with a diagnosis of co-occurring autism are significantly more likely to show self-injury, aggression and disruption to the environment whilst individuals with deficits in receptive and expressive communication are significantly more likely to show self-injury, but overall, the meta-analysis highlighted the paucity of methodologically robust studies of risk markers for challenging behaviours in populations with intellectual disabilities (McClintock et al. 2003).

A more recent systematic review identified 38 studies investigating association of challenging behaviours with 11 behavioural, psychiatric and psychosocial factors (van den Akker et al. 2020), but association with physical conditions has been less investigated despite the fact that epidemiological studies show that patients with intellectual disabilities have more physical conditions than the general population (e.g. Cooper et al. 2015). A systematic review of studies investigating the association of physical conditions with challenging behaviours in population with intellectual disabilities identified 45 studies, which looked at general medical conditions, motor impairment, epilepsy, sensory impairment, gastrointestinal disease, sleep disorders, dementia and other conditions. Among the identified studies, there were only four high-quality and seven well-conducted observational studies. Significant and independent associations were found for challenging behaviours and urinary incontinence, pain related to cerebral palsy and chronic sleep problems, and between self-injurious behaviour and visual impairment. No association was found with hearing impairment, bowel incontinence, mobility impairment or epilepsy. Many other physical conditions were not addressed at all (de Winter et al. 2011).

Furthermore, contradicting findings on epilepsy were reported in a systematic review by Deb et al. (2020), who after sensitivity analysis, found a significantly higher rate of overall challenging behaviours in the epilepsy group compared with the non-epilepsy group in a meta-analysis of 10 studies, but the effect size was very small (0.16). Aggression and self-injurious behaviour both showed a statistically significant higher rate in the epilepsy group, with very small effect sizes (0.16 and 0.28 respectively). No significant intergroup difference was observed in the rate of stereotypy (Deb et al. 2020).

Study type

Hypothesis testing

Study design

Retrospective cohort study

Feasibility counts

Records on all eligible patients with intellectual disabilities between the period of 1 January 2009 and 31 December 2019 will be analysed. The feasibility of identifying patients with intellectual disabilities in CPRD data has been successfully tested in previous research (Carey et al. 2016; 2017; Glover et al. 2019; 2020).

Carey et al. (2016) searched for any code used by the NHS Primary Care Quality and Outcomes Framework for learning disability and codes for conditions usually associated with intellectual disabilities (as is planned in the current project), such as chromosomal and metabolic disorders. This approach identified 21,859 adults (aged 18 years) registered in 451 English practices for at least 1 day between 1 January 2009 and 31 March 2013. The cross-sectional analysis reported on a subset of 408 practices that were providing high-quality data on 1 January 2012. A total of 14,751 people with intellectual disabilities aged 18–84 years who had been registered for at least 30 days on the 1 January 2012 date were included, along with 86,221 matched controls. Since our study will have a longer follow-up time (1 January 2009-31 December 2019) and will include patients with intellectual disabilities of all ages as opposed to adults only as has been done in Carey et al. (2016), we expect our sample size to be larger and, thus, have sufficient statistical power.

In the second study by Carey and colleagues (2017), 21,859 adults with intellectual disabilities, registered during 2009–13, were initially identified using an extended list of Read codes for intellectual disabilities and associated conditions. Specific analyses were based on smaller subgroups of adults with intellectual disabilities: a cross-sectional analysis of health and health-care quality on 1 January 2012 (n = 14,751), a longitudinal analysis of mortality and hospital admissions during 2009–13 (n = 16,666) and individual health checks (n = 7,510). Since our study will have a longer follow-up time (1 January 2009-31 December 2019) and will include patients with intellectual disabilities of all ages as opposed to adults only as has been done in Carey et al. (2017), we expect our sample size to be larger and, thus, have sufficient statistical power.

Glover and colleagues (2019; 2020) identified people with intellectual disabilities by searching for any code used by the NHS Primary Care Quality and Outcomes Framework for learning disabilities, and codes for specific syndromes such as Down syndrome. This search identified 59,279 patients (aged 0-85+) with intellectual disabilities registered in English practices for at least 1 day between 1 April 2010 and 31 March 2014. As the proposed study will analyse data from 1 January 2009 up until 31 December 2019, the sample will likely be larger than in the cited study, and, thus, will have sufficient statistical power.

If we assume a 0.5% prevalence of intellectual disabilities in people of all ages as per findings from the Glover et al. (2019; 2020) study using a CPRD GOLD database with a follow-up period of April 2010-March 2014, then within the population of currently registered research acceptable (de-duplicated) patients (N=16,305,791 in total; N=3,020,680 for GOLD database and N=13,285,111 for Aurum database), 81,529 patients would be expected to have intellectual disabilities on any given day. Further sample size considerations are discussed in the next section.

Sample size considerations

As per details provided in the 'Feasibility counts' section, if we assume a 0.5% prevalence of intellectual disabilities as per findings reported by Glover et al. (2019; 2020) using a CPRD GOLD database with a follow-up period of April 2010-March 2014, then within the population of currently registered research acceptable (de-duplicated) patients (N=16,305,791 in total; N=3,020,680 for GOLD database and N=13,285,111 for Aurum database) 81,529 patients would be expected to have intellectual disabilities on any given day. Prevalence studies of challenging behaviours in nationwide primary care samples are lacking, but if we assume that a prevalence of challenging behaviours in a population of people of all ages with intellectual disabilities in England is as described in a study of seven District Health Authorities in the UK by Emerson et al. (2001), 16.5% (N=13,452) of patients with intellectual disabilities are expected to have any form of challenging behaviours on any given day during the study follow-up period of 2009-2019, with prevalence rates for specific types of challenging behaviours expected to be much higher (e.g., 42.0% for aggressive behaviours). Given the estimated sample size, the study is expected to have sufficient statistical power. References after amendments: Emerson, E., Kiernan, C., Alborz, A., Reeves, D., Mason, H., Swarbrick, R., Mason, L., & Hatton, C. (2001). The prevalence of challenging behaviours: A total population study. Research in Developmental Disabilities, 22(1), 77-93.

Planned use of linked data and benefit to patients in England and Wales

Our analysis will use the Clinical Practice Research Datalink (CPRD) GOLD and Aurum databases which will be anonymised. Patients' GP records will be linked with:
1. HES Admitted Patient Care to test for interaction effects with the effect modifier of physical conditions;
2. Mental Health Services Data Set (MHSDS), available for the period of April 2007-November 2015, to assess any attenuation in the associations between the mental comorbidities moderator and challenging behaviours;
3. Practice and Patient Level Index of Multiple Deprivation to adjust for confounding effects of socioeconomic deprivation for all analyses.

Importance/impact: This project will extend the existing work on challenging behaviours in people with intellectual disabilities by using nationwide CPRD data to investigate the incidence of challenging behaviours in a large nationwide population of primary care patients with intellectual disabilities in England. Using stratified Cox proportional hazard models, we will also examine the association between challenging behaviours and physical conditions (i.e. pain, sleep problems, constipation, epilepsy, urinary/bowel incontinence and sensory impairments) in this population, including exploring how different health (e.g. severity of intellectual disabilities, co-occurring autism/genetic syndromes/mental comorbidities), demographic (e.g. age, gender, ethnicity) and socioeconomic (e.g. Practice/Patient Level Index of Multiple Deprivation) factors may affect this relationship. The proposed study will provide a much-needed foundation for future research and intervention development designed to reduce health inequalities experienced by patients with intellectual disabilities through better management of challenging behaviours and improved understanding of their association with physical health problems in this population. This is particularly important in the light of lack of existing evidence in this area of research.

Dissemination: We will adopt a diverse strategy for dissemination to ensure that study outputs are useful, timely, appropriate and action oriented. These will include reports targeted at particular audiences (e.g. individuals and families affected by intellectual disabilities and challenging behaviours, health and social care practitioners and commissioners, policy makers and academics). We will publish in peer reviewed scientific journals and disseminate findings via the University of Glasgow, Warwick, Central Lancashire and St George's, University of London websites, newsletters and social media channels.
Study results will also be presented and discussed with NHS Education England and Scotland, Health Education and Improvement Wales, Departments of Health and Social Care in England and Wales, Wales Challenging Behaviour Community of Practice, Healthcare Improvement Scotland, Scottish Government's Mental Health Directorate and Cross-Party Disability Group and at conferences relevant to frontline clinicians (i.e. Royal Colleges of Psychiatrists and General Practitioners and Public Health England conferences). We will also disseminate findings to the authors of the NICE guideline NG11 'Challenging behaviour and learning disabilities: prevention and interventions for people with learning disabilities whose behaviour challenges', the British Psychological Society's Clinical Practice Guidelines 'Psychological interventions for severely challenging behaviours shown by people with learning disabilities' and the Welsh Government's Learning Disability Improving Lives Programme.

Definition of the study population

Data on all patients with intellectual disabilities will be acquired from CPRD. Individuals with intellectual disabilities will be identified using Read codes in Appendix 1. Co-occurring challenging behaviours will be defined as a binary variable (present or not) based on READ codes in Appendix 1. Patients registered in a practice between January 1 January 2009 and 31st December 2019 will be included in the study. We require that their practice allows linkage to HES, MHSDS and IMD data, resulting in the inclusion of English practices. Patients who are registered at CPRD practices not participating in the linkage scheme, or who have individually opted out, will not be included.

Earliest cohort entry date will be 1/1/2009. Patients who register after 1/1/2009, or who are diagnosed with intellectual disabilities during the study period, will be followed from that date. Patients will be censored if they transfer out from their CPRD practices, or opt out before the end of the study period (31/12/2019). However, challenging behaviour diagnoses of patients who leave a CPRD practice prior to the end of the study period will be included in a sensitivity analysis.

Index case for a case would be one of the following:
• 1/1/2009 (if a patient is actively registered on this date with a diagnosis of challenging behaviours prior to 2009)
• date of diagnosis (if a patient’s date of first diagnosis of challenging behaviours is after 1/1/2009 while they are registered)
• date of registration (if a patient’s date of registration is after 1/1/2009 and they have a prior diagnosis to that registration)

Inclusion criteria:
• all patients with intellectual disabilities (as defined in READ codes in Appendix 1) registered at a general practice for at least six months prior to cohort entry date
• patients who receive a diagnosis of intellectual disabilities after cohort entry date and have a valid registration status for at least six months

Exclusion criteria
• patients registered at practices not participating in the linkage scheme, or who have individually opted out

Selection of comparison groups/controls

Not applicable

Exposures, outcomes and covariates

Exposure: record of intellectual disabilities

The exposure variable will be a record of intellectual disabilities. Patients with intellectual disabilities will be identified using searches for any code included in Appendix 1, as has been done previously with CPRD (Carey et al. 2016; 2017).

Primary outcome: challenging behaviours
Outcome variables will be presence of challenging behaviours, which will be identified by Read codes in Appendix 1 (Sheehan et al. 2015;).

Moderator: record of co-occurring physical conditions
Patients with intellectual disabilities and co-occurring physical conditions (i.e. pain, sleep problems, constipation, epilepsy, urinary/bowel incontinence and sensory impairments) will be identified using searches for any relevant READ code, as has been done previously with CPRD (e.g. Glover et al. 2017; Hosking et al. 2016).

Mediators:
severity of intellectual disabilities, co-occurring autism/genetic syndromes/mental comorbidities will be identified using searches for any code included in Appendix 1 (e.g. Glover et al. 2017; Hosking et al. 2016; Houghton et al. 2018)

Confounding covariates:
Demographic (age, gender, ethnicity) and socioeconomic (Practice/Patient Level Index of Multiple Deprivation) factors

Data/statistical analysis

The analysis will be performed using STATA/SPSS Software. In our main analyses, patients will be stratified into subgroups by age, sex and co-occurring intellectual disabilities. All of the analyses as described below will be performed for each subgroup.

Phase 1
We will calculate the incidence of challenging behaviours within the group with intellectual disabilities. Our binary outcome will be defined on the basis of having a record of a relevant Read code for co-occurring challenging behaviours (Appendix 1). Records will be stratified by age, sex, severity of intellectual disabilities and/or co-occurring physical conditions/autism/genetic syndromes/mental comorbidities if cell sizes allow. Tables of baseline characteristics will be described for all patients with intellectual disabilities. For continuous data, mean, median, range, and standard deviations will be calculated. For categorical variables, frequency and percentages will be reported.

Phase 2
The main statistical analyses will be conducted using stratified Cox proportional hazard models (Sjolander & Greenland 2013). The Cox models will be run univariately, then multivariably. The multivariable analysis will comprise (i) adjustment for demographic (age, gender, ethnicity) and socioeconomic (Practice/Patient Level Index of Multiple Deprivation) factors, (ii) the addition of mediators (i.e. severity of intellectual disabilities, co-occurring autism/genetic syndromes/mental comorbidities) factors) to the above model to assess any attenuation in the associations (iii) the use of likelihood ratio tests to test for interaction effects with the effect modifier (i.e. physical conditions including pain, sleep problems, constipation, epilepsy, urinary/bowel incontinence and sensory impairments) if there is sufficient statistical power and if found to be statistically significant, further sub-group analyses. We will test for proportional hazards in the Cox models using both graphical methods and a formal statistical test based on Scheonfeld residuals. Where the assumption of proportional hazards is violated, we will augment the models with time varying covariates. To account for patients with multiple events over the 10-year study period and investigate incidence rate ratios, we will conduct a Poisson regression, similarly to the methodology applied by Sheehan et al. (2015).

Plan for addressing confounding

We plan to adjust for the following confounding factors: sex, age, ethnicity, socioeconomic status using the Practice/Patient Level Index of Multiple Deprivation.

Plans for addressing missing data

We will use complete case analysis for missing data for all demographic analyses. As a sensitivity analysis we will compare results using complete case analysis with results derived using multiple imputation with chained equations. The number of imputed datasets will be determined through the Fraction of Missing Information (FMI) statistic. Both sets of analyses will be reported.

Patient or user group involvement

No patients will be involved in this research at this stage. There are no plans to disseminate this proposal to patients' groups at this stage.

Plans for disseminating & communicating

The authors are planning to disseminate the study results through peer-reviewed journals, academic conference presentations, the Scottish Government, key stakeholders and policy makers communication channels, the Scottish Learning Disabilities Observatory website, newsletter and social media. There are no restrictions on the extent and timing of publication.

Conflict of interest statement

None

Limitations of study design

Practice-based deprivation measure may not represent individual-level socioeconomic status. Information on the socioeconomic status of patients is available for English practices only, which may limit the generalisability of our findings. There may also be an under-representation of people with intellectual disabilities and co-occurring challenging behaviours in the dataset, as there may be a significant number of patients without a formal diagnosis. Errors and inconsistencies in recording challenging behaviours may present problems in data quality. There may also be unrecorded or unreported physical conditions in the population with intellectual disabilities in the primary care data. In investigating the association of specific physical conditions with challenging behaviours, we may be limited to analysing more common physical conditions only due to small sub-sample sizes. Variation in coding across different practices and over time may introduce systematic biases. Linkage to the HES Admitted Patient Care and Mental Health Services Data Set (MHSDS) may limit the sample size, depending on the quality of the linked data.

References

Bowring, D. L., Painter, J., & Hastings, R. P. (2019). Prevalence of challenging behaviour in adults with intellectual disabilities, correlates, and association with mental health. Current Developmental Disorders Reports, 6(4), 173-181.

Branford, D., Gerrard, D., Saleem, N., Shaw, C., & Webster, A. (2019). Stopping over-medication of people with intellectual disability, autism or both (STOMP) in England Part 1–history and background of STOMP. Advances in Mental Health and Intellectual Disabilities 13(1), 31-40.

Carey, I. M., Shah, S. M., Hosking, F. J., DeWilde, S., Harris, T., Beighton, C., & Cook, D. G. (2016). Health characteristics and consultation patterns of people with intellectual disability: a cross-sectional database study in English general practice. British Journal of General Practice, 66(645), e264-e270.

Carey, I. M., Hosking, F. J., Harris, T., DeWilde, S., Beighton, C., & Cook, D. G. (2017). An evaluation of the effectiveness of annual health checks and quality of health care for adults with intellectual disability: An observational study using a primary care database. Health Services and Delivery Research, 5(25), 1-170.

Cooper, S.-A., McLean, G., Guthrie, B., McConnachie, A., Mercer, S., Sullivan, F., & Morrison, J. (2015). Multiple physical and mental health comorbidity in adults with intellectual disabilities: Population-based cross-sectional analysis. BMC Family Practice, 16(1), 110–120.

de Winter, C. F., Jansen, A. A. C., & Evenhuis, H. M. (2011). Physical conditions and challenging behaviour in people with intellectual disability: A systematic review. Journal of Intellectual Disability Research, 55(7), 675-698.

Deb, S., Brizard, B. A., & Limbu, B. (2020). Association between epilepsy and challenging behaviour in adults with intellectual disabilities: Systematic review and meta-analysis. BJPsych Open, 6(5), e114-e114.

Glover, G., Williams, R., Heslop, P., Oyinlola, J., & Grey, J. (2017). Mortality in people with intellectual disabilities in England. Journal of Intellectual Disability Research, 61(1), 62-74.

Glover, G., Williams, R., Tompkins, G., & Oyinlola, J. (2019). An observational study of the use of acute hospital care by people with intellectual disabilities in England. Journal of Intellectual Disability Research, 63(2), 85– 99.

Glover, G., Williams, R., & Oyinlola, J. (2020). An observational cohort study of numbers and causes of preventable general hospital admissions in people with and without intellectual disabilities in England. Journal of Intellectual Disability Research, 64(5), 331-344.

Hosking, F., Harris, T., DeWilde, S., Beighton, C., Shah, S., Cook, D., & Carey, I. (2016). Do health checks for adults with intellectual disability reduce emergency hospital admissions? Evaluation of a natural experiment. Journal of Epidemiology and Community Health (1979), 70(Suppl 1), A11-A12.

Houghton, R., Liu, C., & Bolognani, F. (2018). Psychiatric comorbidities and psychotropic medication use in autism: A matched cohort study with ADHD and general population comparator groups in the United Kingdom: Psychotropic medication use in autism in the UK. Autism Research, 11(12), 1690-1700.

McClintock, K., Hall, S., & Oliver, C. (2003). Risk markers associated with challenging behaviours in people with intellectual disabilities: A meta-analytic study. Journal of Intellectual Disability Research, 47(6), 405-416.

Sheehan, R., Hassiotis, A., Walters, K., Osborn, D., Strydom, A., & Horsfall, L. (2015). Mental illness, challenging behaviour, and psychotropic drug prescribing in people with intellectual disability: UK population based cohort study. BMJ: British Medical Journal, 351, h4326-h4326.

Sjolander, A. & Greenland, S. (2013). Ignoring the matching variables in cohort studies – when is it valid and why? Statistics in Medicine, 32(27), 4696-4708.

van den Akker, N., Kroezen, M., Wieland, J., Pasma, A., & Wolkorte, R. (2020). Behavioural,

psychiatric and psychosocial factors associated with aggressive behaviour in adults with intellectual disabilities: A systematic review and narrative analysis. Journal of Applied Research in Intellectual Disabilities, 00, 1-63.

Appendices

appendix-1_0.pdf

Grant ID
