# Supplementary material for: Temporal associations between incident physical health problems/sensory impairments and challenging behaviours in people with intellectual disabilities: a population-based longitudinal cohort study of primary care in England
Source: BMJ Open. 2026 Jul 3;16(7):e111117. doi: 10.1136/bmjopen-2025-111117 (PMC13343113; doi:10.1136/bmjopen-2025-111117)
Supplement: online supplemental file 4 [file bmjopen-16-7-s004.docx]

**Table S2. Cox Proportional Hazards Regression Models using 1 Month Time-Window**

| Predictor | Unadjusted | | Demographically Adjusted | | Fully Adjusted | |
| --- | --- | --- | --- | --- | --- | --- |
|  | HR | p-value | HR | p-value | HR | p-value |
| Constipation | 2.045 (1.850 - 2.261) | <.001*** | 1.789 (1.613 - 1.984) | <.001*** | 1.710 (1.551 - 1.885) | <.001*** |
| Epilepsy | 1.684 (1.557 - 1.820) | <.001*** | 1.588 (1.453 - 1.736) | <.001*** | 1.612 (1.487 - 1.747) | <.001*** |
| Pain | 1.296 (1.208 - 1.389) | <.001*** | 1.155 (1.066 - 1.251) | <.001*** | 1.103 (1.027 - 1.184) | 0.007** |
| Visual Impairment | 1.788 (1.648 - 1.940) | <.001*** | 1.645 (1.503 - 1.801) | <.001*** | 1.645 (1.511 - 1.791) | <.001*** |
| Hearing Impairment | 1.930 (1.622 - 2.296) | <.001*** | 1.705 (1.537 - 1.891) | <.001*** | 1.752 (1.587 - 1.933) | <.001*** |
| Bowel Incontinence | 2.395 (2.137 - 2.683) | <.001*** | 2.190 (1.910 - 2.510) | <.001*** | 2.199 (1.926 - 2.511) | <.001*** |
| Urinary Incontinence | 2.340 (2.075 - 2.639) | <.001*** | 2.064 (1.849 - 2.304) | <.001*** | 2.000 (1.804 - 2.218) | <.001*** |
| Sleep Problems | 2.080 (1.883 - 2.298) | <.001*** | 2.046 (1.805 - 2.318) | <.001*** | 1.968 (1.759 - 2.202) | <.001*** |

**Table S3. Cox Proportional Hazards Regression Models using 3 Month Time-Window**

| Predictor | Unadjusted | | Demographically Adjusted | | Fully Adjusted | |
| --- | --- | --- | --- | --- | --- | --- |
|  | HR | p-value | HR | p-value | HR | p-value |
| Constipation | 2.185 (1.996 - 2.393) | <.001*** | 1.950 (1.776 - 2.140) | <.001*** | 1.832 (1.684 - 1.993) | <.001*** |
| Epilepsy | 1.681 (1.554 - 1.818) | <.001*** | 1.615 (1.472 - 1.773) | <.001*** | 1.628 (1.497 - 1.770) | <.001*** |
| Pain | 1.457 (1.363 - 1.557) | <.001*** | 1.312 (1.214 - 1.418) | <.001*** | 1.234 (1.155 - 1.319) | <.001*** |
| Visual Impairment | 1.820 (1.673 - 1.980) | <.001*** | 1.664 (1.516 - 1.826) | <.001*** | 1.652 (1.516 - 1.800) | <.001*** |
| Hearing Impairment | 2.029 (1.723 - 2.391) | <.001*** | 1.771 (1.595 - 1.968) | <.001*** | 1.805 (1.634 - 1.993) | <.001*** |
| Bowel Incontinence | 2.481 (2.222 - 2.772) | <.001*** | 2.277 (1.990 - 2.606) | <.001*** | 2.264 (1.990 - 2.575) | <.001*** |
| Urinary Incontinence | 2.404 (2.151 - 2.686) | <.001*** | 2.146 (1.930 - 2.385) | <.001*** | 2.052 (1.862 - 2.260) | <.001*** |
| Sleep Problems | 2.085 (1.902 - 2.286) | <.001*** | 2.037 (1.815 - 2.286) | <.001*** | 1.928 (1.742 - 2.133) | <.001*** |

**Table S4. Cox Proportional Hazards Regression Models using 6 Month Time-Window**

| Predictor | Unadjusted | | Demographically Adjusted | | Fully Adjusted | |
| --- | --- | --- | --- | --- | --- | --- |
|  | HR | p-value | HR | p-value | HR | p-value |
| Constipation | 2.246 (2.068 - 2.439) | <.001*** | 2.037 (1.859 - 2.232) | <.001*** | 1.885 (1.738 - 2.044) | <.001*** |
| Epilepsy | 1.637 (1.511 - 1.774) | <.001*** | 1.582 (1.438 - 1.741) | <.001*** | 1.572 (1.444 - 1.712) | <.001*** |
| Pain | 1.539 (1.439 - 1.646) | <.001*** | 1.405 (1.294 - 1.525) | <.001*** | 1.301 (1.214 - 1.395) | <.001*** |
| Visual Impairment | 1.835 (1.687 - 1.996) | <.001*** | 1.686 (1.534 - 1.853) | <.001*** | 1.656 (1.520 - 1.804) | <.001*** |
| Hearing Impairment | 2.021 (1.735 - 2.353) | <.001*** | 1.792 (1.610 - 1.995) | <.001*** | 1.793 (1.622 - 1.981) | <.001*** |
| Bowel Incontinence | 2.618 (2.336 - 2.933) | <.001*** | 2.360 (2.069 - 2.691) | <.001*** | 2.313 (2.043 - 2.618) | <.001*** |
| Urinary Incontinence | 2.378 (2.147 - 2.634) | <.001*** | 2.123 (1.915 - 2.353) | <.001*** | 2.002 (1.826 - 2.196) | <.001*** |
| Sleep Problems | 2.032 (1.866 - 2.213) | <.001*** | 1.954 (1.752 - 2.179) | <.001*** | 1.818 (1.655 - 1.998) | <.001*** |

Note. * p < 0.05, ** p < 0.01, *** p < 0.001. Demographically adjusted models included age as a predictor, and were stratified by sex, ethnicity, and IMD. Fully adjusted models included the demographic adjustments, as well as diagnoses of anxiety, bipolar disorder, major depression, and schizophrenia as predictors, and were additionally stratified by diagnoses of autism.
