# Supplementary material for: Temporal associations between incident physical health problems/sensory impairments and challenging behaviours in people with intellectual disabilities: a population-based longitudinal cohort study of primary care in England
Source: BMJ Open. 2026 Jul 3;16(7):e111117. doi: 10.1136/bmjopen-2025-111117 (PMC13343113; doi:10.1136/bmjopen-2025-111117)
Supplement: online supplemental file 5 [file bmjopen-16-7-s005.docx]

Little’s MCAR test was statistically significant (χ2(30) = 4343.66, p <0.001) suggesting that the data are not Missing Completely At Random.

**Table S5. Logistic regression model predicting missingness of severity of Intellectual Disability**

| Variable | Odds Ratio | 95% CI | p-value |
| --- | --- | --- | --- |
| **Male** | 0.97 | (0.94, 1) | 0.02755 |
| **Ethnicity – ref: White** |  |  |  |
| Asian | 1.27 | (1.21, 1.33) | < 0.001 |
| Black | 1.39 | (1.32, 1.47) | < 0.001 |
| Multiple | 1.40 | (1.29, 1.52) | < 0.001 |
| Other | 1.78 | (1.58, 2.01) | < 0.001 |
| **GP practice level IMD – ref: 1** |  |  |  |
| 2 | 0.94 | (0.89, 0.99) | 0.02707 |
| 3 | 0.93 | (0.88, 0.98) | 0.00765 |
| 4 | 1.15 | (1.1, 1.22) | < 0.001 |
| 5 | 1.13 | (1.08, 1.19) | < 0.001 |
| **Autism** | 0.66 | (0.64, 0.69) | < 0.001 |

**Table S6. Logistic regression model predicting missingness of ethnicity**

| Variable | Odds Ratio | 95% CI | p-value |
| --- | --- | --- | --- |
| **Male** | 1.03 | (1, 1.07) | 0.0791 |
| **Intellectual Disability Severity – ref: Mild** |  |  |  |
| Moderate | 1.00 | (0.96, 1.04) | 0.9239 |
| Severe | 1.01 | (0.96, 1.06) | 0.7239 |
| Profound | 1.04 | (0.92, 1.17) | 0.5268 |
| **GP practice level IMD – ref: 1** |  |  |  |
| 2 | 0.94 | (0.88, 1.01) | 0.0900 |
| 3 | 0.88 | (0.83, 0.94) | <0.001 |
| 4 | 0.73 | (0.69, 0.78) | <0.001 |
| 5 | 0.64 | (0.6, 0.68) | <0.001 |
| **Autism** | 0.99 | (0.95, 1.04) | 0.6675 |
