# Supplementary material for: Temporal associations between incident physical health problems/sensory impairments and challenging behaviours in people with intellectual disabilities: a population-based longitudinal cohort study of primary care in England
Source: BMJ Open. 2026 Jul 3;16(7):e111117. doi: 10.1136/bmjopen-2025-111117 (PMC13343113; doi:10.1136/bmjopen-2025-111117)
Supplement: online supplemental file 6 [file bmjopen-16-7-s006.docx]

## **Sub-sample of participants with data on level of intellectual disabilities**

**Table S7. Demographic characteristics of cohort.**

| Demographics | n | Proportion of Sample |
| --- | --- | --- |
| **Sex** | |  |
| Female | 20,082 | 40.70% |
| Male | 29,265 | 59.30% |
| **Ethnicity** | |  |
| White | 22,809 | 46.22% |
| Asian | 2,688 | 5.45% |
| Black | 1,960 | 3.97% |
| Multiple | 801 | 1.62% |
| Other | 353 | 0.72% |
| Unknown | 20,736 | 42.02% |
| **IMD** | |  |
| 1 | 6,197 | 12.56% |
| 2 | 8,192 | 16.60% |
| 3 | 10,385 | 21.04% |
| 4 | 11,269 | 22.84% |
| 5 | 13,258 | 26.87% |
| Unknown | 46 | 0.09% |
| **Physical health issues/ sensory impairments** | |  |
| Constipation | 8,257 | 16.73% |
| Epilepsy | 11,218 | 22.73% |
| Pain | 22,280 | 45.15% |
| Visual Impairment | 7,775 | 15.76% |
| Hearing Impairment | 3,309 | 6.71% |
| Bowel Incontinence | 2,047 | 4.15% |
| Urinary Incontinence | 4,808 | 9.74% |
| Sleep Problems | 13,276 | 26.90% |
| **Mental Health Conditions** |  |  |
| Anxiety | 5,109 | 10.35% |
| Bipolar Disorder | 727 | 1.47% |
| Major Depression | 4,100 | 8.31% |
| Schizophrenia | 1,871 | 3.79% |
| **ID level** | |  |
| Mild | 18,263 | 37.01% |
| Moderate | 17,593 | 35.65% |
| Severe | 12,329 | 24.98% |
| Profound | 1,162 | 2.35% |
| **Autism Spectrum Conditions** | 10,894 | 22.08% |

**Table S8. Incidence of challenging behaviours stratified by subgroups.**

|  |  | | Challenging Behaviour Events | Person Years | Rate Per Person Year |
| --- | --- | --- | --- | --- | --- |
| **Sex** | |  | | | |
| Female |  | | 18,138 | 142,680 | 0.13 |
| Male |  | | 24,925 | 195,765 | 0.13 |
| **Age Group** | |  | | | |
| <18 |  | | 5,098 | 39,698 | 0.13 |
| 18-29 |  | | 11,353 | 83,468 | 0.14 |
| 30-39 |  | | 7,056 | 58,965 | 0.12 |
| 40-49 |  | | 8,911 | 75,912 | 0.12 |
| 50-59 |  | | 6,274 | 49,366 | 0.13 |
| 60-69 |  | | 3,207 | 24,099 | 0.13 |
| 70-79 |  | | 1,003 | 6,202 | 0.16 |
| 80+ |  | | 161 | 735 | 0.22 |
| **Ethnicity** | |  | | | |
| White |  | | 22,878 | 183,238 | 0.12 |
| Asian |  | | 2,821 | 23,118 | 0.12 |
| Black |  | | 1,761 | 13,148 | 0.13 |
| Multiple |  | | 750 | 5,764 | 0.13 |
| Other |  | | 307 | 2,343 | 0.13 |
| **IMD** | |  | | | |
| 1 |  | | 4,783 | 37,673 | 0.13 |
| 2 |  | | 7,098 | 56,977 | 0.12 |
| 3 |  | | 9,360 | 74,310 | 0.13 |
| 4 |  | | 10,189 | 79,525 | 0.13 |
| 5 |  | | 11,601 | 89,723 | 0.13 |
| **ID level** | |  | | | |
| Mild |  | | 12,594 | 92,751 | 0.14 |
| Moderate |  | | 15,250 | 121,537 | 0.12 |
| Severe |  | | 13,894 | 112,960 | 0.12 |
| Profound |  | | 1,325 | 11,197 | 0.12 |
| **Autism** | |  | | | |
| No |  | | 31,487 | 251,200 | 0.12 |
| Yes |  | | 11,576 | 87,245 | 0.13 |

**Table S9. Incidence of clinical diagnoses over study period by challenging behaviours in primary care records.**

| Clinical Diagnoses | Challenging Behaviour Present | | | No Challenging Behaviour | | |
| --- | --- | --- | --- | --- | --- | --- |
|  | Events | Incidence | n (%) | Events | Incidence | n (%) |
| Constipation | 12,333 | 0.11 | 4,094 (27.5%) | 10,911 | 0.08 | 4,105 (18.6%) |
| Epilepsy | 45,966 | 0.42 | 4,851 (32.6%) | 47,617 | 0.36 | 6,217 (28.2%) |
| Pain | 41,655 | 0.38 | 9,199 (61.9%) | 49,887 | 0.38 | 12,990 (58.9%) |
| Visual Impairment | 7,485 | 0.07 | 3,637 (24.5%) | 7,708 | 0.06 | 3,979 (18.0%) |
| Hearing Impairment | 3,630 | 0.03 | 1,577 (10.6%) | 3,125 | 0.02 | 1,641 (7.4%) |
| Bowel Incontinence | 2,532 | 0.02 | 1,222 (8.2%) | 1,213 | 0.01 | 798 (3.6%) |
| Urinary Incontinence | 5,805 | 0.05 | 2,562 (17.2%) | 4,376 | 0.03 | 2,181 (9.9%) |
| Sleep Problems | 22,397 | 0.21 | 6,659 (44.8%) | 18,814 | 0.14 | 6,474 (29.3%) |

*Note.* Incidence is presented as events per person year.

**Table S10. Cox Proportional Hazards Regression Models**

| Predictor | Unadjusted | | Demographically Adjusted | | Fully Adjusted | |
| --- | --- | --- | --- | --- | --- | --- |
|  | HR | p-value | HR | p-value | HR | p-value |
| Constipation | 1.854 (1.713 - 2.007) | <.001*** | 1.742 (1.580 - 1.920) | <.001*** | 1.581 (1.433 - 1.744) | <.001*** |
| Epilepsy | 1.329 (1.247 - 1.416) | <.001*** | 1.272 (1.184 - 1.367) | <.001*** | 1.207 (1.128 - 1.291) | <.001*** |
| Pain | 1.317 (1.241 - 1.397) | <.001*** | 1.213 (1.142 - 1.288) | <.001*** | 1.160 (1.090 - 1.233) | <.001*** |
| Visual Impairment | 1.363 (1.259 - 1.475) | <.001*** | 1.314 (1.195 - 1.445) | <.001*** | 1.273 (1.161 - 1.396) | <.001*** |
| Hearing Impairment | 1.642 (1.440 - 1.872) | <.001*** | 1.515 (1.372 - 1.673) | <.001*** | 1.452 (1.317 - 1.601) | <.001*** |
| Bowel Incontinence | 1.986 (1.795 - 2.198) | <.001*** | 1.895 (1.681 - 2.135) | <.001*** | 1.720 (1.542 - 1.919) | <.001*** |
| Urinary Incontinence | 1.713 (1.559 - 1.881) | <.001*** | 1.622 (1.470 - 1.789) | <.001*** | 1.461 (1.329 - 1.606) | <.001*** |
| Sleep Problems | 1.709 (1.584 - 1.843) | <.001*** | 1.618 (1.488 - 1.759) | <.001*** | 1.512 (1.389 - 1.647) | <.001*** |

*Note. * p < 0.05, ** p < 0.01, *** p < 0.001. Demographically adjusted models included age as a predictor, and were stratified by sex, ethnicity, and IMD. Fully adjusted models included the demographic adjustments, as well as diagnoses of anxiety, bipolar disorder, major depression, and schizophrenia as predictors, and were additionally stratified by diagnoses of autism.*
