# Supplementary material for: Temporal associations between incident physical health problems/sensory impairments and challenging behaviours in people with intellectual disabilities: a population-based longitudinal cohort study of primary care in England
Source: BMJ Open. 2026 Jul 3;16(7):e111117. doi: 10.1136/bmjopen-2025-111117 (PMC13343113; doi:10.1136/bmjopen-2025-111117)
Supplement: online supplemental file 7 [file bmjopen-16-7-s007.docx]

# **Analyses using imputed data for ethnicity, socioeconomic deprivation and level of intellectual disabilities**

| Demographics | n | Proportion of Sample |
| --- | --- | --- |
| **Sex** | | |
| Female | 69,297 | 41.50% |
| Male | 97,692 | 58.50% |
| **Ethnicity** | | |
| White | 128,197 | 76.77% |
| Asian | 16,987 | 10.17% |
| Black | 13,409 | 8.03% |
| Multiple | 5,449 | 3.26% |
| Other | 2,947 | 1.76% |
| **IMD** | | |
| 1 | 21,421 | 12.83% |
| 2 | 25,974 | 15.55% |
| 3 | 31,780 | 19.03% |
| 4 | 39,874 | 23.88% |
| 5 | 47,940 | 28.71% |
| **Physical Health Conditions** | | |
| Constipation | 20,989 | 12.57% |
| Epilepsy | 26,519 | 15.88% |
| Pain | 66,855 | 40.04% |
| Visual Impairment | 12,869 | 7.71% |
| Hearing Impairment | 6,829 | 4.09% |
| Bowel Incontinence | 4,067 | 2.44% |
| Urinary Incontinence | 11,180 | 6.70% |
| Sleep Problems | 34,240 | 20.50% |
| **Mental Health Conditions** | | |
| Anxiety | 14,534 | 8.70% |
| Bipolar Disorder | 1,619 | 0.97% |
| Major Depression | 13,027 | 7.80% |
| Schizophrenia | 4,857 | 2.91% |
| **ID level** | | |
| Mild | 65,919 | 39.48% |
| Moderate | 59,468 | 35.61% |
| Severe | 37,812 | 22.64% |
| Profound | 3,790 | 2.27% |
| **Autism Spectrum Conditions** | 28,699 | 17.19% |

**Table S11. Demographic characteristics of cohort**

##

**Table S12. Challenging behaviours stratified by subgroups.**

|  | Challenging Behaviour Events | Person Years | Rate Per Person Year |
| --- | --- | --- | --- |
| **Sex** | | | |
| Female | 36,063 | 270,911 | 0.13 |
| Male | 51,875 | 389,005 | 0.13 |
| **Age Group** | | | |
| <18 | 18,944 | 139,574 | 0.14 |
| 18-29 | 24,070 | 180,101 | 0.13 |
| 30-39 | 12,152 | 90,936 | 0.13 |
| 40-49 | 13,246 | 100,194 | 0.13 |
| 50-59 | 9,640 | 73,625 | 0.13 |
| 60-69 | 6,125 | 47,215 | 0.13 |
| 70-79 | 2,689 | 20,221 | 0.13 |
| 80+ | 1,072 | 8,051 | 0.13 |
| **Ethnicity** | | | |
| White | 68,190 | 513,530 | 0.13 |
| Asian | 9,013 | 66,807 | 0.14 |
| Black | 6,898 | 51,339 | 0.13 |
| Multiple | 2,535 | 18,594 | 0.14 |
| Other | 1,302 | 9,646 | 0.14 |
| **IMD** | | | |
| 1 | 10,781 | 81,490 | 0.13 |
| 2 | 13,870 | 102,505 | 0.14 |
| 3 | 17,062 | 128,550 | 0.13 |
| 4 | 20,684 | 155,973 | 0.13 |
| 5 | 25,541 | 191,398 | 0.13 |
| **ID level** | | | |
| Mild | 34,160 | 255,999 | 0.13 |
| Moderate | 31,081 | 234,507 | 0.13 |
| Severe | 20,768 | 154,952 | 0.13 |
| Profound | 1,929 | 14,459 | 0.13 |
| **Autism** | | | |
| No | 70,691 | 531,838 | 0.13 |
| Yes | 17,247 | 128,079 | 0.14 |

##

**Table S13. Cox Proportional Hazards Regression Models**

| Predictor | Unadjusted | | Demographically Adjusted | | Fully Adjusted | |
| --- | --- | --- | --- | --- | --- | --- |
|  | HR | p-value | HR | p-value | HR | p-value |
| Constipation | 2.273 (2.141 - 2.414) | <.001*** | 2.259 (2.128 - 2.398) | <.001*** | 2.073 (1.948 - 2.205) | <.001*** |
| Epilepsy | 1.691 (1.607 - 1.780) | <.001*** | 1.687 (1.603 - 1.775) | <.001*** | 1.651 (1.570 - 1.737) | <.001*** |
| Hearing Impairment | 1.964 (1.810 - 2.132) | <.001*** | 1.955 (1.800 - 2.123) | <.001*** | 1.866 (1.717 - 2.029) | <.001*** |
| Visual Impairment | 1.856 (1.760 - 1.957) | <.001*** | 1.857 (1.762 - 1.958) | <.001*** | 1.740 (1.651 - 1.834) | <.001*** |
| Bowel Incontinence | 2.543 (2.368 - 2.731) | <.001*** | 2.548 (2.376 - 2.733) | <.001*** | 2.437 (2.272 - 2.615) | <.001*** |
| Urinary Incontinence | 2.310 (2.193 - 2.433) | <.001*** | 2.306 (2.189 - 2.429) | <.001*** | 2.127 (2.016 - 2.244) | <.001*** |
| Pain | 1.596 (1.534 - 1.662) | <.001*** | 1.590 (1.527 - 1.655) | <.001*** | 1.429 (1.367 - 1.494) | <.001*** |
| Sleep Problems | 1.992 (1.919 - 2.067) | <.001*** | 1.984 (1.911 - 2.059) | <.001*** | 1.797 (1.730 - 1.866) | <.001*** |

*Note. * p < 0.05, ** p < 0.01, *** p < 0.001. Demographically adjusted models included age as a predictor, and were stratified by sex, ethnicity, and IMD. Fully adjusted models included the demographic adjustments, as well as diagnoses of anxiety, bipolar disorder, major depression, and schizophrenia as predictors, and were additionally stratified by diagnoses of autism.*
