# Supplementary material for: Temporal associations between incident physical health problems/sensory impairments and challenging behaviours in people with intellectual disabilities: a population-based longitudinal cohort study of primary care in England
Source: BMJ Open. 2026 Jul 3;16(7):e111117. doi: 10.1136/bmjopen-2025-111117 (PMC13343113; doi:10.1136/bmjopen-2025-111117)
Supplement: Supplementary file 8 [file bmjopen-2025-111117.reviewer_comments.pdf]

## PEER REVIEW HISTORY

BMJ Open publishes all reviews undertaken for accepted manuscripts. Reviewers are asked to complete a checklist review form and are provided with free text boxes to elaborate on their assessment. These free text comments are reproduced below.

### ARTICLE DETAILS

**Title (Provisional)**

Temporal associations between incident physical health problems/ sensory impairments and challenging behaviours in people with intellectual disabilities: a population-based longitudinal cohort study of primary-care in England

**Authors**

Millington, Elliot; Rydzewska, Ewelina; Jahoda, Andrew; Hastings, Richard P.; MacDonald, Anne; Fleming, Michael; Richards, Caroline; Gillooly, Amanda; Smiley, Elita; Chauhan, Umesh; Pacatti, Christine; Melville, Craig

---

### VERSION 1 - REVIEW

---

| | |
|---|---|
| **Reviewer** | **1** |
| **Name** | **Gangadharan, Satheesh** |
| **Affiliation** | **Loughborough University Loughborough School of Design and Creative Arts, Design school** |
| **Date** | **19-Oct-2025** |
| **COI** | **None** |

---

This research is focused on an important area and drawing data from primary care records, covers a large population for a long period of time. 166,989 people with ID are identified and included.

A clear and short list of physical health conditions and sensory impairments selected from the relationship already established in literature but replicated in a much large data base.

Authors have discussed the limitations which include the coding issue- probably under identifying conditions and the problems of missing data.

Study is well designed and analysed.

I feel however authors could explain a further issue with the data recording in primary care, that is the time at which code appears on the data base may not equate to the time the condition has started. If we take behaviours for example, it is possible behaviour is coded when an individual presents with behaviour (which is the assumption made in the paper) or when an individual presents with a physical health problem and the GP or nurse note the

presence of behaviour as well. Behaviour may have existed for a long time but only picked up and recorded at that point.

Including a long time period (12 months) before and after the time of diagnosis of physical health condition gives a good window to overcome the above but still does not completely avoid it.

Poor data quality related to the degree of LD is also another significant issue but this has been mentioned in the weakness.

A minor format error- Page 18- line 10- word "similar" is repeated.

---

| | |
|---|---|
| **Reviewer** | **2** |
| **Name** | **Czajkowski, Marek** |
| **Affiliation** | **Örebro University, School of Medical Sciences** |
| **Date** | **28-Oct-2025** |
| **COI** | **None** |

---

General Comments

This study uses unique UK primary care data to validate previous findings on whether physical health problems/ sensory impairments can act as biological vulnerability factors and increase the risk of challenging behaviours in people with intellectual disabilities, by adding temporal dimension to the association. The pure association of individual physical health problems with challenging behaviours has been previously described in evidence syntheses as referred to in this publication.

Therefore, the temporal aspect is the most interesting one and requires equal attention.

o The study's aims and rationale need clearer articulation, particularly regarding the temporal relationship being investigated.

o The rationale for using a 12-month time window before and after the challenging behaviour to identify physical health problems or sensory impairments in order to assess temporal associations should be better justified.

o Intuitively, a shorter window (e.g., 2–8 weeks, 3 months) may be more appropriate—particularly for self-limiting conditions such as pain or constipation—and could be considered for use in a sensitivity analysis to assess the robustness of the temporal association.

o A sensitivity analysis of the chosen time window before, until the challenging behaviour only could also be considered.

o A title adjustment could be also considered: "Temporal Associations between incident physical health problems/ sensory impairments and challenging behaviours in people with

intellectual disabilities: a population-based longitudinal cohort study in primary-care”. This better reflects the study’s focus and avoids ambiguity in the original title.

Specific comments

General

References throughout the manuscript should be placed before punctuation marks (e.g., full stops, commas) and within the same sentence, not at the beginning of a new sentence.

Introduction

Page 4, line 42

Remove redundancy from the aim-sentence ”act as biological vulnerability factors and increase the risk of”, e.g. ”act as biological vulnerability factors for challenging behaviours.”

P6, l1–8

The sentence “A systematic review of…” has an unclear subsentence due to ambiguous use of “which.” Try rephrasing. Additionally, the reference is missing and should be included at the end of the first sentence, not at the end of paragraph.

P6, l36–40

The statement regarding observational studies should specify the type of validity being discussed. Do you mean a threat to internal validity (due to not reflecting true causal relationships), or the strength of the evidence (due to retrospective design)?

P7, l1-4

The second research question requires some justification in the introduction. If the hypothesis is that physical health problems are risk factors, why is the study also examining the 12 months after their onset? Alternatively move such nuances to the method section. I find the actual explanation in methods not sufficient for the 24 months time window.

P4, l26–27

Consider removing or moving the paragraph break after the sentence: “This framework for understanding challenging behaviours is reproduced in Figure 1 below,” and than introduce the Figure 1.

Figure 1

I am unable to assess whether reproducing the original Figure 1 from Bowring et al. is acceptable by BMJ Open guidelines or sufficiently reported. Please consult the editorial staff.

Methods

P7, l52-53

Read codes should be written with capital ”R” and were used untill 2018. SNOMED CT was adopted thereafter by NHS England as far as I know. Consider explaining how sample was selected between 2018 and 31/12/2019?

The presentation of Read codes in the appendix would benefit from clearer categorization, e.g. codes used for identifying:

- Intellectual disabilities,

- Primary outcome: challenging behaviours

- Covariates: autism, genetic syndromes, mental comorbidities, etc.

P8, l6

The full study protocol is difficult to locate online. A part of it is available here: ” https://www.cprd.com/approved-studies/association-physical-conditions-challenging-behaviours-people-intellectual” Consider providing a refference with direct hyperlink or clearer instructions for access to the full-protocol that is disclosed.

P8, l29

Clarify whether epilepsy is treated as a incident of seizure or a chronic condition. Consider specifying “epileptic seizures” if referring to clinical events.

P9, l5

The phrase “record of a diagnosis” may be misleading due to especially for conditions like pain, which are symptoms rather than diagnoses. Consider using “recorded code” or “reported clinical event” instead.

P11, l 37

The explanation of overlapping exposure periods is unclear; consider rephrasing it for clarity.

The criteria for applying the Andersen–Gill extension of the Cox model assumes that recurrent events are treated as independent, each sharing a common baseline hazard rate across all events for a subject.

I do not have sufficient expertise to judge whether exposure stacking or incident stacking, which result in shorter time periods, is the appropriate approach for the chosen analysis. Consultation with a statistician can be considered.

P9, l16-… and l49-…

The paragraph “The challenges surrounding…” appears twice with minor differences. Consider consolidating or removing the duplicate.

Results

In general, providing a descriptive explanation of the most important findings in each table would help guide the reader through the key results. E.g. explanation of how to understand Cox proportional hazard models with adjustments for the highest ratio.

P18, l10

Word ”similar” appears to be repeated. Please remove the duplicate.

Discussion

p19, l27

Replace “eight” with “seven” if that is the correct number.

P21,l11

The long time window discussed in the beginning may introduce indirectness. Consider discussing this limitation and its implications for the the temporal association.

P21,l48

The scope of the implications “This can only be achieved by the provision of well-resourced, multidisciplinary, specialist intellectual disabilities services and effective partnership working across primary, secondary and tertiary healthcare services.” is too broad given that the study focuses on primary care settings. Consider revising or removing.

Conclusion

Adding a clear concluding statement should be considered.

P77–192

These pages contain content that is difficult to scrutinize. Consider improving formatting.

---

| | |
|---|---|
| **Reviewer** | **3** |
| **Name** | **Kelson, Zoe** |
| **Affiliation** | **University of Exeter, Mathematics** |
| **Date** | **03-Dec-2025** |
| **COI** | **None** |

---

This retrospective, population-based cohort study aims to determine whether the onset of physical health problems/ sensory impairments is associated with incident challenging behaviours.

Reviewer comments:

Can the modelling methods applied please also be briefly specified in the Abstract?

"UK primary care data sourced from the Clinical Practice Research Datalink (CPRD) Aurum and GOLD databases, covering over 850,000 person-years between 2009 and 2019...

166,989 individuals with recorded intellectual disabilities were included in the cohort "

and

"This study used primary care data from the Aurum and GOLD databases maintained by the Clinical Practice Research Datalink (CPRD)... CPRD contains data from participating GP practices in the UK linked to GP practice level Index of Multiple Deprivation...

15.29% of UK practices participate in the CPRD Aurum, which served 19.75% of the UK population as current patients at the time of extraction. CPRD GOLD contained 4.40% of practices and 4.52% of the UK population."

and

"This study included records from 166,989 people identified as having intellectual disabilities who collectively contributed 854,133 person-years or a mean average of 5.8 years per person. The mean age of the cohort at entry to the study was 33.1 (SD 19.4). Further demographic characteristics of this cohort can be found in Table 1"

Can the authors please further comment on whether the analysed cohort can be considered to be demographically and characteristically representative?

"This paper describes a retrospective cohort study of people with intellectual disabilities, using data between 01/01/2009 and 31/12/2019. "

Can the authors please comment on whether this cohort can be considered to be up-to-date? Might things have changed since 2019?

"For descriptive analyses, patients were categorised by age at study entry into 8 groups (<18, 18-29, 30-39, 40-49, 50-59, 60-69, 70-79, and 80+ years). For inferential analyses, age was treated as a continuous variable."

Can age please also be described as a continuous variable?

"hazard ratios were estimated using the Andersen-Gill extension of the Cox proportional hazards model with separate models fitted for each individual physical health condition...

The models were adjusted for demographic variables (age, sex, ethnicity, deprivation and the presence of an autism spectrum condition) and then a final model was adjusted for demographic variables and incident mental health diagnoses treated as time-varying covariates using the same methodology as physical health conditions, with each mental health condition modelled separately with exposure periods extending 12 months before and after each diagnosis date. Violations of the assumption of proportional hazards were assessed using a combination of formal tests of Scheonfeld residuals and graphical methods. In response to these violations, models which accounted for demographic variables were stratified by sex, ethnicity, deprivation and the presence of an autism spectrum condition. "

Appropriate modelling methods have been applied by the authors.

"All models accounted for clustering at the practice level and within the person."

Can the authors please clarify how clustering has been adjusted for (e.g. applying robust standard errors or multilevel modelling)?

"Complete records were requested for any person with a record of intellectual disabilities in the primary care data, as determined using read codes (see codes used in the supplementary materials). "

and

"Each analysis presented here were conducted using a complete case approach that only includes participants with no missing data"

Can the authors please assess whether missing data can be assumed to be missing at random?

"As a sensitivity analysis, modelling was completed that included only participants with available data on severity of intellectual disabilities (supplemental file 2). As a second sensitivity analysis, an alternative dataset was created using multiple imputation with chained equations (MICE) for those with missing data for their ethnicity, socioeconomic deprivation and severity of intellectual disabilities. The participants' GP practice, sex, ethnicity, socioeconomic deprivation of their local area, age at study entry, and presence of the diagnostic outcomes in this study were each used to inform the imputation of missing data for ethnicity, socioeconomic deprivation and severity of intellectual disabilities."

The authors do well to conduct sensitivity analyses on the approach taken for handling missing data.

"A small number of people with indeterminate sex were present in the data but were excluded from the analysis to reduce disclosure risks."

Can the authors please specify how many is a 'small number of people' here?

"Primary care coding likely under-identifies challenging behaviours as only the most severe cases are seen in clinical settings • Over 70% of patients had missing data on intellectual disability severity" [strengths and limitations of this study]

and

"Despite these strengths, several limitations must be acknowledged. First, coding in primary care is likely to under-identify both challenging behaviours because it is only people experiencing the most severe challenging behaviours who will have been assessed in primary care or seen by specialist services for people with intellectual disabilities. Second, over 70% of the cohort had missing data on severity of intellectual disability. Although the sensitivity analyses performed to take account of these missing data did not result in a significant change in the findings (although the strength of associations was attenuated), improving the quality of data on severity of intellectual disability recorded in primary care settings, and in other datasets, would increase the rigour of future longitudinal studies investigating the health needs of people with intellectual disabilities. Furthermore, while our

design allows for inferences about temporality compared to cross-sectional studies, the observational nature of the data precludes definitive causal conclusions"

The authors have provided a discussion on the study limitations.

Thanks for providing a copy of the study protocol and STROBE checklist.

---

## VERSION 1 - AUTHOR RESPONSE

**Prof. Gangadharan**

*I feel however authors could explain a further issue with the data recording in primary care, that is the time at which code appears on the data base may not equate to the time the condition has started. If we take behaviours for example, it is possible behaviour is coded when an individual presents with behaviour (which is the assumption made in the paper) or when an individual presents with a physical health problem and the GP or nurse note the presence of behaviour as well. Behaviour may have existed for a long time but only picked up and recorded at that point. Including a long time period (12 months) before and after the time of diagnosis of physical health condition gives a good window to overcome the above but still does not completely avoid it.*

A sentence has been added to the limitations section highlighting this concern.

*A minor format error- Page 18- line 10- word "similar" is repeated.*

The additional similar has been removed.

**Mr. Czajkowski**

*The study's aims and rationale need clearer articulation, particularly regarding the temporal relationship being investigated.*

We have expanded the articulation of the aims and rationale for the study throughout the introduction and methods sections. Further explanations of the temporal relationship being investigated have been added, and supplemented with sensitivity analyses using one, three and six month time windows.

*The rationale for using a 12-month time window before and after the challenging behaviour to identify physical health problems or sensory impairments in order to assess temporal associations should be better justified.*

The justifications for the time window choice has been consolidated into a single paragraph in the data analyses section, supplemented with further detail, and sensitivity analyses provided.

*Intuitively, a shorter window (e.g., 2–8 weeks, 3 months) may be more appropriate—particularly for self-limiting conditions such as pain or constipation—and could be considered for use in a sensitivity analysis to assess the robustness of the temporal association.*

A sensitivity analysis comparing the results from alternative time windows has been included in the supplementary materials.

*A sensitivity analysis of the chosen time window before, until the challenging behaviour only could also be considered.*

Upon consideration, we believe that only considering the period prior to the challenging behaviour would not benefit the analysis. Our reasoning for this is that there are high proportion of cases where challenging behaviours are issue that leads to consultation with primary care, leading to them being recorded first, while the physical health condition is then recorded later following investigation. By only including the first section of the window, these relevant cases would not be accounted for.

*A title adjustment could be also considered: "Temporal Associations between incident physical health problems/ sensory impairments and challenging behaviours in people with intellectual disabilities: a population-based longitudinal cohort study in primary-care". This better reflects the study's focus and avoids ambiguity in the original title.*

The title has been adjusted following the reviewer's suggestion.

*References throughout the manuscript should be placed before punctuation marks (e.g., full stops, commas) and within the same sentence, not at the beginning of a new sentence.*

In-text citations have been moved to before punctuation.

*Page 4, line 42 Remove redundancy from the aim-sentence "act as biological vulnerability factors and increase the risk of", e.g. "act as biological vulnerability factors for challenging behaviours."*

Edited to the reviewer's suggestion.

*P6, l1–8 The sentence "A systematic review of..." has an unclear subsentence due to ambiguous use of "which." Try rephrasing. Additionally, the reference is missing and should be included at the end of the first sentence, not at the end of paragraph.*

This sentence was broken into two separate sentences to make the meaning clearer.

*P6, l36–40 The statement regarding observational studies should specify the type of validity being discussed. Do you mean a threat to internal validity (due to not reflecting true causal relationships), or the strength of the evidence (due to retrospective design)?*

This sentence was clarified to mean internal validity.

*P7, l1-4 The second research question requires some justification in the introduction. If the hypothesis is that physical health problems are risk factors, why is the study also examining the 12 months after their onset? Alternatively move such nuances to the method section. I find the actual explanation in methods not sufficient for the 24 months time window.*

We have further justified the use of the 12 month window before and after the onset of physical health problems/ sensory impairments in the methods section. However, the sensitivity analyses using one, three and six month time windows before and after the onset of physical health problems/ sensory impairments show that the relationships persist regardless of the time window used, and add to the strength of the findings.

*P4, l26–27 Consider removing or moving the paragraph break after the sentence: "This framework for understanding challenging behaviours is reproduced in Figure 1 below," and than introduce the Figure 1.*

The sentence introducing figure 1 has been moved to the end of the previous paragraph.

*Figure 1 I am unable to assess whether reproducing the original Figure 1 from Bowring et al. is acceptable by BMJ Open guidelines or sufficiently reported. Please consult the editorial staff.*

We discussed this with the editorial team, and a hyperlink to the conditions of the licence has been added.

*P8, l6 The full study protocol is difficult to locate online. A part of it is available here: " https://www.cprd.com/approved-studies/association-physical-conditions-challenging-behaviours-people-intellectual" Consider providing a refference with direct hyperlink or clearer instructions for access to the full-protocol that is disclosed.*

Edited the study registration section to signpost that the full protocol is included as part of the supplementary materials.

*P8, l29 Clarify whether epilepsy is treated as a incident of seizure or a chronic condition. Consider specifying "epileptic seizures" if referring to clinical events.*

Text has been added to the methods section to clarify that, in keeping with previous studies, we are using clinical diagnoses of epilepsy, rather than seizure events which have a much broader aetiology.

*P9, l5 The phrase "record of a diagnosis" may be misleading due to especially for conditions like pain, which are symptoms rather than diagnoses. Consider using "recorded code" or "reported clinical event" instead.*

The word "diagnosis" has been changed to "clinical event" or similar where appropriate.

*P11, l 37 The explanation of overlapping exposure periods is unclear; consider rephrasing it for clarity. The criteria for applying the Andersen–Gill extension of the Cox model assumes that recurrent events are treated as independent, each sharing a common baseline hazard rate across all events for a subject. I do not have sufficient expertise to judge whether exposure stacking or incident stacking, which result in shorter time periods, is the appropriate approach for the chosen analysis. Consultation with a statistician can be considered.*

The description of the model in the data analysis section has been redrafted with the intention of offering greater clarity. In consultation with the statistician on our project team, we believe the Andersen-Gill model to be sufficient for these data. We appreciate and understand the reviewer's concern, however, we believe that the use of robust variance estimation accommodates for correlations between recurrent events and the impacts of using shorter intervals to represent overlapping exposure intervals.

*P9, l16-... and l49-... The paragraph "The challenges surrounding..." appears twice with minor differences. Consider consolidating or removing the duplicate.*

The second of these paragraphs has been removed.

*Results. In general, providing a descriptive explanation of the most important findings in each table would help guide the reader through the key results. E.g. explanation of how to understand Cox proportional hazard models with adjustments for the highest ratio.*

We have added text explanations of the results of each table

*P18, l10 Word "similar" appears to be repeated. Please remove the duplicate.*

The second "similar" has been removed.

p19, l27. Replace “eight” with “seven” if that is the correct number.
We believe eight to be the correct number of conditions considered in the study. If there are any sections of the manuscript where this is inconsistent, we would be pleased to have them highlighted.

*P21,l11 The long time window discussed in the beginning may introduce indirectness. Consider discussing this limitation and its implications for the the temporal association.*

We have stated that the use of the four time windows has added to the validity of the findings and strengthened the study.

*P21,l48 The scope of the implications “This can only be achieved by the provision of well-resourced, multidisciplinary, specialist intellectual disabilities services and effective partnership working across primary, secondary and tertiary healthcare services.” is too broad given that the study focuses on primary care settings. Consider revising or removing.*

This section has been expanded to explain what this needs to look like if services are going to more effectively manage the complex and frequent relationships between physical health problems/ sensory impairments and challenging behaviours.

*Conclusion. Adding a clear concluding statement should be considered.*

A conclusions section has been added to the revised manuscript.

*P77–192. These pages contain content that is difficult to scrutinize. Consider improving formatting.*

We recognise that supplemental file 5 is not processed in a way that makes it easy to scrutinise when using the auto-generated PDFs. This data has been uploaded to the system as a .csv so that it is easily reusable by other researchers, and should be viewable if opened in a spreadsheeting software.

**Prof. Kelson**

*Can the modelling methods applied please also be briefly specified in the Abstract?*

A sentence was added to the design section of the abstract to describe the modelling technique.

*Can the authors please further comment on whether the analysed cohort can be considered to be demographically and characteristically representative?"*

A sentence has been added to the data source section describing prior research looking at the representativeness of CPRD for the general population. It is more challenging to assess the representativeness of the intellectual disability cohort specifically as prior work has used CPRD itself, and England does not have good administrative data to compare to.

*Can the authors please comment on whether this cohort can be considered to be up-to-date? Might things have changed since 2019?*

We have added a comment in the limitations section that recognises that there is no data for the past five years, and reflected on different positive and negative effects that this may have.

*Can age please also be described as a continuous variable?*

In the current version of the manuscript, the continuous age characteristics of the cohort are described in the "Cohort Members" section of the results.

*Can the authors please clarify how clustering has been adjusted for (e.g. applying robust standard errors or multilevel modelling)?*

This sentence was modified to clarify that the clustering was applied using robust variance estimates.

*Can the authors please assess whether missing data can be assumed to be missing at random?*

A couple of sentences have been added to the methods describing how we assessed that the data was missing at random.

*Can the authors please specify how many is a 'small number of people' here?*

This sentence has been modified to clarify that under 5 people with indeterminate sex were present, ensuring that the small size is clearer without identifying the exact number.

---

## VERSION 2 - REVIEW

---

| | |
|---|---|
| **Reviewer** | **2** |
| **Name** | **Czajkowski, Marek** |
| **Affiliation** | **Örebro University, School of Medical Sciences** |
| **Date** | **26-Mar-2026** |
| **COI** | |

---

Thank you for comments and the revised manuscript.

Addressing your response to my comment:

"p19, l27. Replace "eight" with "seven" if that is the correct number.

We believe eight to be the correct number of conditions considered in the study. If there are any sections of the manuscript where this is inconsistent, we would be pleased to have them highlighted."

Response: I agree that eight is the correct number of conditions. The confusion likely arises from how they were listed (e.g., in the abstract – "constipation, epilepsy, pain, visual and hearing impairment, bowel and urinary incontinence and sleep problems."). Therefore, "seven" should be replaced with "eight" in the indicated sentences.

P18 L52 – "New episodes of all seven of the physical health problems/ sensory impairments…"

P16 L5 – " …before and after the onset of all seven physical health problems/ sensory impairments."

I have no further comments.

| | |
|---|---|
| **Reviewer** | **3** |
| **Name** | **Kelson, Zoe** |
| **Affiliation** | **University of Exeter, Mathematics** |
| **Date** | **12-Mar-2026** |
| **COI** | |

Thanks to the authors for their responses and revisons.

Article revision:

"Patterns of missingness were assessed by comparing the demographic and clinical characteristics between those individuals with and without complete data. These analyses indicated that missingness was associated with several observed covariates, consistent with the data being Missing At Random. "

Reviewer request:

In addition to stating this important assessment in the article, can the authors please show this analyiss of missing data in the supplementary material?

## VERSION 2 - AUTHOR RESPONSE

Prof. Kelson
In addition to stating this important assessment in the article, can the authors please show this analyiss of missing data in the supplementary material?

To evidence the analyses of missing data, we have added a supplementary file 5 which details the MCAR test to show that data were not missing completely at random and logistic regressions which used to justify the choice of imputation variables. The manuscript has been modified to flag this file when talking about missingness.

Mr. Czajkowski
I agree that eight is the correct number of conditions. The confusion likely arises from how they were listed (e.g., in the abstract – "constipation, epilepsy, pain, visual and hearing impairment, bowel and urinary incontinence and sleep problems."). Therefore, "seven" should be replaced with "eight" in the indicated sentences.
P18 L52 – "New episodes of all seven of the physical health problems/ sensory impairments…"
P16 L5 – " …before and after the onset of all seven physical health problems/ sensory impairments."

We now have a clearer idea about where the confusion has arisen from, we have made a number of changes to the wording of the manuscript. We made the distinction between the incontinences and sensory impairments in the abstract and changed "seven" to "eight" in the highlighted sections.
